# Lipid Biomarkers From Microbial Mats on the McMurdo Ice Shelf, Antarctica: Signatures for Life in the Cryosphere

**DOI:** 10.3389/fmicb.2022.903621

**Published:** 2022-06-10

**Authors:** Thomas W. Evans, Maria J. Kalambokidis, Anne D. Jungblut, Jasmin L. Millar, Thorsten Bauersachs, Hendrik Grotheer, Tyler J. Mackey, Ian Hawes, Roger E. Summons

**Affiliations:** ^1^Department of Earth, Atmospheric, and Planetary Sciences, Massachusetts Institute of Technology, Cambridge, MA, United States; ^2^Life Sciences Department, Natural History Museum, London, United Kingdom; ^3^School of Earth and Environmental Sciences, Cardiff University, Cardiff, United Kingdom; ^4^Institute of Geosciences, Christian-Albrechts-University of Kiel, Kiel, Germany; ^5^Marine Geochemistry, Alfred Wegener Institute, Helmholtz Centre for Polar and Marine Research, Bremerhaven, Germany; ^6^Coastal Marine Field Station, University of Waikato, Tauranga, New Zealand

**Keywords:** cyanobacteria, Antarctica, intact polar lipid, heterocyte glycolipids, bacteriohopanepolyol, microbial mats, homeoviscous adaptation

## Abstract

Persistent cold temperatures, a paucity of nutrients, freeze-thaw cycles, and the strongly seasonal light regime make Antarctica one of Earth’s least hospitable surface environments for complex life. Cyanobacteria, however, are well-adapted to such conditions and are often the dominant primary producers in Antarctic inland water environments. In particular, the network of meltwater ponds on the ‘dirty ice’ of the McMurdo Ice Shelf is an ecosystem with extensive cyanobacteria-dominated microbial mat accumulations. This study investigated intact polar lipids (IPLs), heterocyte glycolipids (HGs), and bacteriohopanepolyols (BHPs) in combination with 16S and 18S rRNA gene diversity in microbial mats of twelve ponds in this unique polar ecosystem. To constrain the effects of nutrient availability, temperature and freeze-thaw cycles on the lipid membrane composition, lipids were compared to stromatolite-forming cyanobacterial mats from ice-covered lakes in the McMurdo Dry Valleys as well as from (sub)tropical regions and hot springs. The 16S rRNA gene compositions of the McMurdo Ice Shelf mats confirm the dominance of Cyanobacteria and Proteobacteria while the 18S rRNA gene composition indicates the presence of Ochrophyta, Chlorophyta, Ciliophora, and other microfauna. IPL analyses revealed a predominantly bacterial community in the meltwater ponds, with archaeal lipids being barely detectable. IPLs are dominated by glycolipids and phospholipids, followed by aminolipids. The high abundance of sugar-bound lipids accords with a predominance of cyanobacterial primary producers. The phosphate-limited samples from the (sub)tropical, hot spring, and Lake Vanda sites revealed a higher abundance of aminolipids compared to those of the nitrogen-limited meltwater ponds, affirming the direct affects that N and P availability have on IPL compositions. The high abundance of polyunsaturated IPLs in the Antarctic microbial mats suggests that these lipids provide an important mechanism to maintain membrane fluidity in cold environments. High abundances of HG keto-ols and HG keto-diols, produced by heterocytous cyanobacteria, further support these findings and reveal a unique distribution compared to those from warmer climates.

## Introduction

Antarctica is replete with environments where complex life is excluded. Sub-zero temperatures, the scarcity of liquid water, nutrient limitation (Sorrell et al., 2013), and strong seasonality with prolonged periods of darkness followed by exposure to high UV radiation present challenges for most organisms (Vincent and Quesada, 1994). While macroscopic eukaryotic life struggles with such extreme conditions, bacteria have been shown to withstand low temperatures and to be well adapted to variable light, nutrient, and osmotic conditions (Jungblut and Neilan, 2010). Cyanobacteria, especially, can proliferate to form dense populations in many aquatic Antarctic environments (Vincent and Quesada, 1994). Notably, the ‘dirty ice’ located on the McMurdo Ice Shelf, located between the Ross Sea and Transantarctic Mountains, supports one of the largest networks of meltwater ponds in Antarctica and these are rich in biomass dominated by mm-cm thick benthic cyanobacteria-based microbial communities, as initially described by Scott in 1905 during his first Antarctic expedition aboard the RRS Discovery (Scott, 1907).

The ‘dirty ice’ region of the McMurdo Ice Shelf is an ablation zone located southeast of Bratina Island and northwest of Southern Victoria Land and comprises an area in excess of 1,500 km^2^ (Howard-Williams et al., 1990). The ice is covered with pockets of dark sediment and other debris derived from the seafloor, which is conveyed to the surface as ice ablates, driven by relatively warm and dry katabatic winds originating from the polar plateau (Debenham, 1919; Glasser et al., 2006). During summer, this area hosts a network of shallow meltwater ponds, varying in size and shape and ranging from 10s to several 10,000s m^2^ (Howard-Williams et al., 1990). Although the water in the ponds share similar sources from combinations of precipitation and melting sea ice, they are characterized by contrasting physicochemical conditions, such as variable salt concentrations, pH-values, and water temperatures (Howard-Williams et al., 1990).

Metagenomic and small subunit rRNA gene studies have confirmed microscopy surveys identifying diverse and highly productive cyanobacterial communities in the ‘dirty ice’ of the McMurdo Ice Shelf (Varin et al., 2012; Jackson et al., 2021). Major taxa comprise the heterocyte forming genera *Anabaena*, *Nostoc*, and *Nodularia* and filamentous non-heterocyte forming genera including *Oscillatoria*, *Phormidium*, *Pseudanabaena*, and *Leptolyngbya* (Jungblut et al., 2005; Varin et al., 2012; Jackson et al., 2021). Accumulation of photosynthetic biomass is reflected by high chlorophyll concentrations of up to 400 mg m^–2^ (Howard-Williams et al., 1990), being in the same range as active and organic-rich intertidal zones of the Wadden Sea (North Sea) (Stal et al., 1985). Other bacterial representatives detected by 16S rRNA gene clone libraries or successfully cultivated from Antarctic microbial mats and sediments include Firmicutes, Actinobacteria, Bacteroidetes, and Proteobacteria (e.g., Sjöling and Cowan, 2003; Varin et al., 2012; Archer et al., 2015). Besides bacterial assemblages, various eukaryotes, such as algae, fungi, and microscopic metazoa, have been reported from Antarctic meltwater ponds (Khan et al., 2011; Jungblut et al., 2012). Due to the high biomass and diverse microbial communities, the McMurdo meltwater ponds are often recognized as microbial oases in an environment that is otherwise hostile to complex life.

These oases are of particular paleobiological interest as they serve as analogs for conditions that prevailed during the Cryogenian Period (∼720 – 636 Ma) when Earth experienced two long-lasting glaciations interspersed with periodic ‘hothouse’ conditions as articulated in the ‘Snowball Earth’ hypothesis (Hoffman et al., 1998, 2017; Rooney et al., 2015). During ice-house times, sea-ice extended to low latitudes, perhaps, completely enveloping the planet. This would have severely limited photosynthesis in the oceans leading to pervasively anoxic water columns (Lechte et al., 2019). Fossil evidence, lipid biomarkers, and geochemical data, however, indicate that biogeochemical cycles were active and that major lineages thrived and diversified during the Cryogenian epoch (Corsetti et al., 2006; Moczydłowska, 2008; Brocks et al., 2017; Hoffman et al., 2017; Johnson et al., 2017). This suggests a prevalence of microbial refugia despite challenging surface conditions. Meltwater pond systems, similar to those of McMurdo Sound, cryoconites, and environments at the edges of the ice shelves could have supplied oxygen-rich meltwaters to the oceans and provided habitable conditions for more complex biota during this period (Hoffman, 2016; Hawes et al., 2018; Lechte et al., 2019).

Lipid biomarkers, such as intact polar lipids (IPLs), heterocyte glycolipids (HGs), and bacteriohopanepolyols (BHPs), have been extensively applied to investigate the chemotaxonomic composition of metabolically active microbial communities. IPLs comprising a polar head group bound to a glycerol backbone and linked to a hydrophobic hydrocarbon tail, encode valuable chemotaxonomic information (Rütters et al., 2002; Sturt et al., 2004). HGs comprise a sugar head group attached to an alkyl chain with 26 – 32 carbon atoms (e.g., Gambacorta et al., 1999; Bauersachs et al., 2009a) and are used to evaluate communities of diazotrophic cyanobacteria (Bauersachs et al., 2011). BHPs are composed of a pentacyclic triterpenoid core with functional groups along a ribose-derived side chain. They are used as biomarkers for bacteria and their ecological niches due to the high diversity of chemical structures and characteristic distribution patterns (e.g., Rashby et al., 2007; Matys et al., 2019).

The high biomass and low anthropogenic interference in these meltwater ponds, coupled with climatic conditions unique to the Antarctic, constitute an ideal natural laboratory to study homeoviscous adaption strategies of the microbial cell membranes. However, to date, only one study has investigated the lipid distribution of actively growing microbial mats in three McMurdo Ice Shelf meltwater ponds (Jungblut et al., 2009), while another recent study investigated lipid biomarkers in ancient microbial mats from the McMurdo Ice Shelf (Lezcano et al., 2022). Here, we expand on this to include comprehensive biomarker analyses combined with 16S and 18S rRNA gene composition data to further characterize the microbial communities that thrive in this unusual setting. We also compare the lipid distribution of the ‘dirty ice’ samples with microbial communities in the ice-covered Antarctic lakes from the McMurdo Dry Valleys and some well-documented (sub)tropical and hot spring environments to assess adaptions of the lipid membrane to changes in nutrient supply, temperature, and irradiance regimes. These findings offer a new perspective on biosignatures of the cryosphere and the Cryogenian Period.

## Materials and Methods

### Sample Collection and Study Sites

**FIGURE 1:**
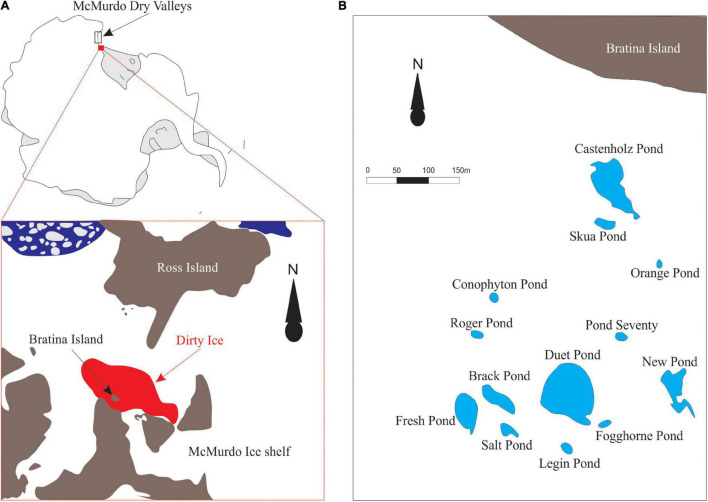
**(A)** The schematic figure of the sampling location on the McMurdo Ice Shelf (78.00’S, 165.30’E). Microbial mats were collected from meltwater ponds on the ‘dirty ice’ marked in red. The figure was modified from Hawes et al. (2018). **(B)** Schematic map of the investigated meltwater ponds in the ‘dirty ice’ (modified from Jackson et al., 2021).

Microbial mat samples were collected from twelve meltwater ponds on the McMurdo Ice Shelf, located adjacent to Bratina Island (Figures 1A,B, 78.00’S, 165.30’E) in January 2018. Clean stainless-steel spatulas were used to excise slices of submerged, intact mats from the pond edges, avoiding the underlying sediment as much as possible. In addition, one semi-dried mat located above the water line was collected from Skua Pond. Layers of different colors were evident in some cases, but not all. However, no delamination was attempted since we were interested to sample the living community throughout. In addition, we collected ‘*Nostoc* spheres’ and a ‘sheet,’ which lay on top of the microbial mats from four ponds (Table 1). The ‘*Nostoc* spheres’ had a size of 5 – 15 mm in diameter, a brownish color, and consisted of a community of *Nostoc* and grazers. They were neutrally buoyant could be scooped up with an aquarium net, thereby avoiding any material from the mats below. They were collected from the littoral zone of the ponds in a water depth, ranging from 10 to 30 cm. All samples were frozen immediately after sample collection in combusted glass jars and returned to New Zealand (NZ), where they were lyophilized, shipped to Cambridge (United States), and then maintained at −20°C until lipid extraction. The physicochemical conditions of the meltwater ponds (Table 1) were measured at each site with a HA40d portable multimeter (Hach, Loveland, CO, United States) (Hawes et al., 1993). Small subsamples of intact mats for DNA analysis were taken with a sterilized scalpel, transferred to sterile Eppendorf tubes, and frozen, on-site, in liquid nitrogen. These samples were initially air-lifted to NZ before being shipped frozen to the Natural History Museum London (United Kingdom), where they were stored at −20°C until further processing.

**TABLE 1:** Investigated meltwater ponds, their physicochemical properties, and the description of the microbial mats in the ponds.

Name	pH	Temperature (°C)	Conductivity (μS cm^–1^)	Description
Legin Pond	9.50	1	2890	Orange colored mat
Fogghorne Pond	9.86	0.5	636	Macroscopic *Nostoc commune* sheet
Pond Seventy	9.88	2.0	3870	Orange colored mat
Salt Pond	9.38	0.0	28400	Green-gray colored mat
Brack Pond	9.27	1.5	6900	Orange-green colored mat
Conophyton Pond	9.24	1.0	1480	Orange colored pinnacle mat
Castenholz Pond	9.55	1.0	2100	Leathery orange colored mat
Orange Pond	9.73	2.5	1541	Green-gray colored mat
Duet Pond	10.16	0.0	119	Thin poorly cohesive gray-green colored mat
Fresh Pond	8.98	0.0	421	Thin orange colored mat
New Pond	NA	NA	NA	Orange pustular mat
Skua Pond	9.69	2.0	1920	Lift-off mat from margin
Legin Pond	9.50	1.0	2890	*Nostoc* sp. spheres
Roger Pond	9.89	2.0	1338	*Nostoc* sp. spheres
Conophyton Pond	9.24	1.0	1480	*Nostoc* sp. spheres

*NA, not available.*

#### Sampling of Microbial Mats From Antarctic Lakes

To evaluate the effect of irradiance on lipid composition, additional mat samples from three ice-covered Antarctic lakes were investigated: Lake Joyce (77.72°S, 161.62°E), Lake Fryxell (77.36°S, 162.6°E) and Lake Vanda (77.52°S, 161.67°E, Supplementary Figure 1). The physicochemical conditions and incident photosynthetically active radiation (PAR) from sample sites are shown in Table 2. All lakes are located in the McMurdo Dry Valleys and are perennially covered with a 4–7 m thick layer of ice that attenuates the photosynthetically active radiation. Samples were collected from depths below the ice-water interface by divers. Samples were either taken using push core or cut from the surrounding mat with a spatula and delaminated from the underlying unconsolidated mat and sediment to maintain mat layering and orientation. Two samples were collected from Lake Joyce: the sample from 20 m depth was sampled from the outer surface of a columnar stromatolite in November 2009 (PAR 0.1%) (Mackey et al., 2018), and the sample from 12 m depth from a flat mat adjacent to a pinnacle stromatolite in November 2014 (PAR 0.7%) (Mackey et al., 2017b). Lake Fryxell samples were collected in November 2012 from 9.1 (PAR 0.5%) and 9.7 (PAR 0.3%) m depth along a benthic transect (Jungblut et al., 2016). Lake Vanda samples (PAR 0.9 – 14%) were collected at 9 m depth in December 2013, where microbial mats grew on former lakeshore deposits, including loose boulders that partially shade underlying mats (Mackey et al., 2017a). For samples from Lakes Joyce, Fryxell, and Vanda, PAR was measured within 1 m of the lakebed by divers using a diver-deployed LI-1400 data logger with an LI-192 cosine corrected underwater quantum detector (LI-COR Biosciences, Lincoln NE, United States). For the partially shaded mats from Lake Vanda, PAR variation across mat samples was modeled using Structure from Motion reconstructions of the lake bottom combined with measurements of the angular light field at the sampling depth (Mackey et al., 2017a). Samples from Lake Vanda were collected across variations in modeled PAR following Matys et al. (2019). A figure of modeled incident PAR and sampling sites is available in Supplementary Figure 2.

**TABLE 2:** Physicochemical conditions in the investigated Antarctic lakes.

Sample	Depth (m)	Temperature (°C)	pH	DO (mg L^–1^)
Lake Vanda	9	4	8.5	∼450^1^
Lake Joyce	12	0.2	10	625^2^
Lake Joyce	20	0.3	8.7	625^2^
Lake Fryxell	9.1	2	7.4	475 – 231^3^
Lake Fryxell	9.7	2	7.5	6^3^

*Dissolved oxygen (DO) concentrations were collected from: Bratina et al. (1998)^2^, Shacat et al. (2004)^1^, and Jungblut et al. (2016)^3^.*

#### Sampling of Microbial Mats From (Sub)tropical and Hot Spring Environments

To compare the lipid composition of the meltwater ponds with microbial mats from (sub)tropical and hot spring environments, additional samples were investigated from Highborne Cay (24.72°N, 76.82°W, Bahamas), Hamelin Pool in Shark Bay (26.26°S, 114.21°E, Australia), and Yellowstone National Park (44.55°N, 110.84°W, “Spent Kleenex-spring,” Lower Geyser Basin). The mats from Highborne Cay were collected in 2010 from a tidal flat and included some encrusted algae. The Hamelin Pool samples were taken from a stromatolite-forming cyanobacterial mat (colloform mat, structures with moderately laminated carbonate fabrics) in June 2011. The Yellowstone sample was collected in June 2012. All samples were stored in sterile Whirl-Paks immediately after sample collection at −20°C and freeze-dried before extraction.

### DNA Extraction and Polymerase Chain Reaction

DNA was extracted using the PowerBiofilm DNA isolation Kit (MO BIO Laboratories). Archaeal and bacterial 16S small ribosomal subunit rRNA (260 bp) was sequenced by the primers utilizing 515 F (5′-TGGCCAGCMGCCGCGGTAA -3′) and 806R (5′- GGACTACNVGGGTWTCTAAT -3′) (Caporaso et al., 2011, 2012). Eukaryotic 18S rRNA gene (130 bp) was amplified using the primers 1391F (5′- GTACACACCGCCCGTC -3′) and EukBr (5′- TGATCCTTCTGCAGGTTCACCTAC -3′) (Amaral-Zettler et al., 2009; Caporaso et al., 2011). Triplicate PCR reactions were prepared in a sterilized laminar hood with a final volume of 20 μL. Different DNA volumes (0.5, 1.0, and 1.5 μL) were used for each replicate to compensate for amplification biases. The reaction mix contained: 9.84 μL sterile PCR-grade water, 0.8 μL of bovine serum albumin (20 mg mL^–1^), 4 μL of 5X GoTaq reaction buffer (Promega), 2 μL of MgCl_2_, 0.16 μL of 200 μM dNTP, 0.2 μL GoTaq G2 DNA polymerase (Promega) and 1 μL of each primer (10 μM). DNA was amplified using the following thermocycler program: 94°C for 3 min, followed by 30 cycles of 94°C for 45 s, 50°C (16S rRNA gene amplification) or 56°C (18S rRNA gene amplification) for 60 s, 72°C for 90 s and a final extension at 72°C for 10 min. In the following, PCR products were purified using AxyPrep Mag PCR Clean-up Kits (Axygen), and triplicate products were combined. The concentration of the purified PCR products was measured in duplicate with a Qubit 2.0 Fluorometer (Thermo Fisher Scientific), and 16S and 18S rRNA gene amplicons were pooled separately at equimolar concentrations for high throughput sequencing. The samples were sequenced (2 × 250 cycles) on an Illumina Miseq platform at the Natural History Museum sequencing facility (United Kingdom). The sequencing data has been deposited at SRA (BioProject ID: PRJNA 758785).

#### 16S and 18S rRNA Gene Sequence Analyses

16S and 18S rRNA gene sequences were analyzed using the QIIME 2 bioinformatics platform, version 2019.4 (Bolyen et al., 2019). The demultiplexed paired-end reads were joined and denoised using DADA2 processing (Callahan et al., 2016). Chimeras and low-quality sequences were removed during this step. Low-frequency amplicon sequence variants (ASVs), which appeared fewer than 3 times, were removed. ASVs were assigned taxonomy using the SILVA 132 database (Quast et al., 2013; Yilmaz et al., 2014). Chloroplast and mitochondrial sequences were also removed, and the datasets were then rarefied to compare community compositions across samples.

### Lipid Extraction

Before lipid extraction, lyophilized mats and ‘*Nostoc* spheres’ were homogenized. IPLs, HGs, and BHPs were extracted four times following a modified Bligh and Dyer protocol (Sturt et al., 2004) with methanol (MeOH), dichloromethane (DCM), and an aqueous solution (2:1:0.8, vol/vol/vol). For the first two extraction steps, the aqueous solution was prepared from monopotassium phosphate (8.7 g L^–1^, pH 7.4), while in steps three and four, trichloroacetic acid (50 g L^–1^, pH 2) was added to the aqueous phase. The sediments were extracted two additional times with DCM and MeOH (9:1, vol/vol) to enhance the extraction efficiency of less polar compounds. The samples were sonicated for 20 min in an ultrasonic bath during each extraction step. The sonicated sediment was then centrifuged at 175 relative centrifugal force (rcf) for 10 min, and the supernatants were combined in a separatory funnel. Deionized Milli-Q water was added to the funnel to allow phase separation. The aqueous solution was extracted three times with DCM. Finally, the organic phase was washed three times with deionized Milli-Q water, and the total lipid extract (TLE) was gently dried under a stream of N_2_ and stored at −20°C until further processing.

#### Intact Polar Lipids and Heterocyte Glycolipids Analyses

The IPLs and HGs were measured in aliquots of the TLE using a 6520 accurate mass quadrupole time-of-flight mass spectrometer (Agilent Technologies) with an electrospray ionization source coupled to a 1200 series high-performance liquid chromatograph (HPLC, Agilent Technologies). IPLs and HGs were separated using a Waters Acquity amide column (150 mm × 2.1 mm, 1.7 μm particle size) used in hydrophilic interaction mode, as described previously (Wörmer et al., 2013). Eluent A consisted of acetonitrile, DCM, formic acid, and ammonium hydroxide (75:25:0.01:0.01, vol/vol/vol/vol), and eluent B was prepared from HPLC-grade water, MeOH, formic acid, and ammonium hydroxide (50:50:0.4:04, vol/vol/vol/vol). The eluent flow and temperature remained constant at 0.4 mL min^–1^ and 40°C throughout the analysis. The gradient started with an isocratic flow of 5% B for 4 min. The proportion of eluent B gradually increased to 25% at 22.5 min and 40% at 26.5 min. Eluent B was held at 40% for 1 min, and then the column was re-equilibrated with 5% B for 8 min. The mass spectrometer was operated in positive ionization mode, scanning 200 – 2,000 daltons. IPLs and HGs were identified based on their exact mass, retention time, and characteristic fragmentation patterns (Sturt et al., 2004; Bauersachs et al., 2009b; Wörmer et al., 2012; Bale et al., 2018). IPL and HG abundances were estimated semi-quantitatively by comparing the parent ion relative to a known amount of injection standard (phosphocholine archaeol, 10 ng injected). For IPLs, response factors were determined from commercially available standards. No standards were available for betaine lipids, ornithine lipids, sulfoquinovosyl diacylglycerol (SQ-DAG), phosphoinositol diacylglycerol (PI-DAG) and HGs, so a relative response factor of unity was chosen for these compounds. Response factors were determined in triplicates with standard variations between 1 and 17% (mean 5%). The lipid measurements are based on singular injections; however, an analytical error of <17% can be applied based on the response factor triplicates. All detected IPLs and HGs were in the linearity range of the instrument.

#### Analysis of Bacteriohopanepolyols

An aliquot of each TLE was acetylated with 40 μL of a mixture of pyridine and acetic anhydride (1:1, vol/vol) at 70°C for 1 h and left overnight at room temperature prior to the analysis of BHPs. The analysis was performed using the same HPLC-MS system and methodology as described by Matys et al. (2017). BHPs were separated by an Agilent Poroshell 120 EC-C19 column (2.1 mm × 150 mm, 2.7 μm particle size), maintained at 30°C, and a solvent flow rate of 0.19 mL min^–1^. Eluent A contained a mixture of MeOH and HPLC-grade water (95:5, vol/vol) and eluent B consisted of isopropanol (100%). The gradient started with 0% B for 2 min, increased to 20% at 20 min, stayed constant for 10 min, and further increased from 20% B (30 min) to 30% B at 40 min. Thereafter, the column was reconditioned for 15 min with 0% B. BHPs were identified based on their accurate mass, retention time, and characteristic fragments (Talbot et al., 2003, 2007, 2008). BHPs were quantified using 3α,12α-dihydroxy-5β-pregnan-20-one 3,12-diacetate (Matys et al., 2017). Due to the highly complex mixture of BHPs in the mat samples and the lack of available standards, BHPs were not corrected for response factors. BHPs were detected in singular injections and were in the linearity range of the instrument. The lipid data has been deposited at the AHED repository (https://doi.org/10.48667/r1zs-v785).

### Calculations

Variation of the microbial community between the different ponds were analyzed by a principal coordinates analysis (PCoA). PCoA analysis for Bacteria, Archaea, Eukaryotes and IPLs was calculated by Bray-Curtis dissimilarities matrices using the “vegan” package in R v. 4.0.4 (Dixon, 2003; R Core Team, 2021) and projected on a 2D-plot.

To evaluate the membrane properties, the double bond index (DBI) and the average chain length (ACL) of IPLs were calculated as described by Evans et al. (2017). C_*X:Y*_ in the DBI refers to the peak area of the compound with x carbon atoms and y unsaturation. The peak area of the compound was multiplied by the number of unsaturation and divided by the total peak area of all compounds (Eq. 1). The ACL was calculated by multiplying the number of x C-atoms in the side chain with the peak area of the compound, divided by the total summed peak area of all compounds (C_*x:y*_, Eq. 2). To account for varying chain lengths of the different IPL species (e.g., PE-DAG C_27 – 38:Y_ and DPG-DAG C_57 – 73:Y_), the number of unsaturations was normalized according to their average chain length (unsaturation per carbon atom, U/C, Eq. 3).


(1) $$\text{DBI}=\frac{\sum y\times C_{x:y}}{\sum C_{x:y}}$$

(2) $$\text{ACL}=\frac{\sum x\times C_{x:y}}{\sum C_{x:y}}$$

(3) $$\text{U}/\text{C}=\frac{D\,B\,I}{A\,C\,L}$$

## Results and Discussion

This study extends our knowledge of bacterial and eukaryotic microbial communities on the McMurdo Ice Shelf by combining lipid biomarker and molecular analyses of microbial mats from twelve meltwater ponds and three separately collected ‘*Nostoc* spheres.’ Prior lipid analysis in these environments screened for hydrocarbons, sterols, and fatty acids (Jungblut et al., 2009; Lezcano et al., 2022). This study focuses on IPLs, HGs, and BHPs to provide insights into how microbes may have shaped their lipid membranes to adapt to the local ice-shelf conditions and, by extension, to environments that may have existed during the Cryogenian glacial epoch.

### 16S and 18S rRNA Gene Diversity in the Meltwater Ponds

**FIGURE 2:**
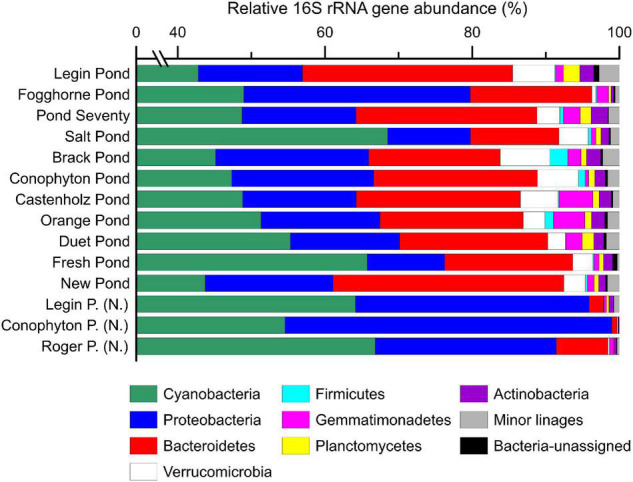
The relative abundance (%) of bacterial 16S rRNA genes in microbial mat communities. Shown are the major bacterial phyla. Samples with sub-fix (N.) indicate ‘Nostoc spheres,’ which were collected in three different meltwater ponds. Detailed results from the 16S rRNA gene sequencing are provided in the supporting information. Note the broken axis between 1 and 39%.

The 16S rRNA gene analysis revealed a dominance of cyanobacterial gene sequences (40–70%, Figure 2), highlighting the predominance of phototrophic bacteria in the microbial mats in the meltwater ponds. Our analysis confirms that cyanobacterial genera, such as *Nostoc, Oscillatoria, Leptolyngbya, Phormidium*, and *Pseudanabaena* (Supplementary Table 1), are important primary producers in Antarctic inland water environments and that they are well adapted to the persistently cold settings (e.g., Jungblut and Neilan, 2010; Jackson et al., 2021). Accordingly, cyanobacteria could have played a major role in sustaining life during the glaciations of the Cryogenian period, consistent with the detection of microbialites preserved in a Cryogenian sequence in Yukon, Canada (Macdonald et al., 2018). Moreover, carbonates from Svalbard, deposited during the Marinoan pan-glaciation (∼650 – 635 Ma ago), show textures consistent with microbial mat accumulation associated with glacial sedimentation (Fairchild et al., 2016), thus, providing strong parallels between Cryogenian sediments and modern deposits from the McMurdo Dry Valley region.

**FIGURE 3:**
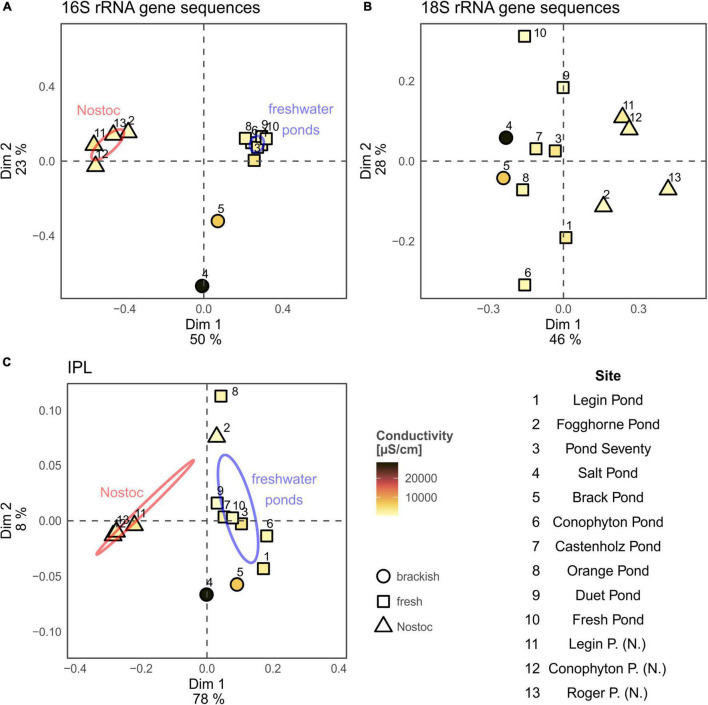
Principal coordinates analysis (PCoA) of the 16S rRNA gene **(A)** and 18S rRNA gene community composition **(B)** and intact polar lipid analysis **(C)** from the microbial mats collected at the McMurdo Ice Shelf. Ellipses for Nostoc type samples (samples 2, 11, 13, and 14; red; see Table 1) and freshwater samples (blue; see Table 1) represent the 95% confidence interval and were calculated using build in function of the “vegan” package. Conductivity results are shown in Table 1. Results from New Pond and Skua Pond were excluded from the PCoA analysis due to missing environmental (New Pond) and rRNA gene sequence data (Skua Pond).

A principal coordinate analysis, based on the Bray–Curtis dissimilarity of 16S rRNA gene bacterial community, revealed distinct assemblages across the different meltwater ponds (Figure 3). The ‘*Nostoc* spheres’ and *Nostoc commune* sheet from Fogghorne Pond formed one cluster, which predominantly contains *Nostoc* spp. (i.e., *N. punctiforme* PCC 73102, Supplementary Table 1) (Rippka et al., 1979). The low conductivity cluster (Figure 3), on the contrary, revealed a higher diversity of cyanobacteria (Supplementary Table 1). The 16S rRNA gene survey from Brack and, in particular, Salt Pond showed variations of the microbial community composition correlating with increasing conductivity (Figure 3) (Jackson et al., 2021). Both ponds showed a high abundance of *Oscillatoria* spp. (Supplementary Table 1), suggesting that these filamentous non-heterocytous cyanobacteria are tolerant of brackish conditions. Moreover, the microbial mats from Salt Pond revealed a prevalence of the heterocytous cyanobacterium *Nodularia*, which is well adapted to intermediate salt concentrations (Möke et al., 2013). High abundances of these cyanobacteria in Salt Pond have also been observed recently by Jackson et al. (2021).

Other important phyla in the microbial mats are Proteobacteria (10–45%) and Bacteroidetes (1–30%) based on 16S rRNA gene sequences (Figure 2). Proteobacteria revealed a high abundance of α*-* and β*-*Proteobacteria, and Bacteroidetes are dominated by Sphingobacteriales and Cytophagales (Supplementary Tables 2, 3). A predominance of these microbes has also been previously found in Antarctic meltwater ponds (Varin et al., 2012; Jackson et al., 2021). Minor groups (<10%) include representatives of Verrucomicrobia, Firmicutes, Gemmatimonadetes, Planctomycetes, and Actinobacteria (Figure 2). This agrees with a previous study that has detected these phyla in the microbial mats from the McMurdo Ice Shelf (Varin et al., 2012). While similar 16S rRNA gene communities were identified for the microbial mats on the McMurdo Ice Shelf and Lake Untersee, we are also aware that PCR can lead to amplification biases (Greco et al., 2020; Jackson et al., 2021). Further evaluation of the composition will benefit from metagenomic approaches that cover all DNA, including ribosomal and protein-coding genes, without PCR.

Archaeal 16S rRNA gene sequences were only detected in Pond Seventy and Salt Pond and represented less than 0.01% of 16S rRNA gene sequences (Supplementary Table 4). This suggests that archaea are only a minor component of microbial mats in meltwater ponds on the McMurdo Ice Shelf, which is in line with previous studies (Varin et al., 2012; Jackson et al., 2021). However, while neither amplicon nor metagenomic analyses of microbial mats on the McMurdo Ice Shelf (Varin et al., 2012; Jackson et al., 2021) have been able to detect high abundances of archaea, it cannot be excluded that the universal 16S rRNA gene primers used here (Caporaso et al., 2012) are less efficient to cover all archaeal groups, as suggested earlier (Parada et al., 2016).

**FIGURE 4:**
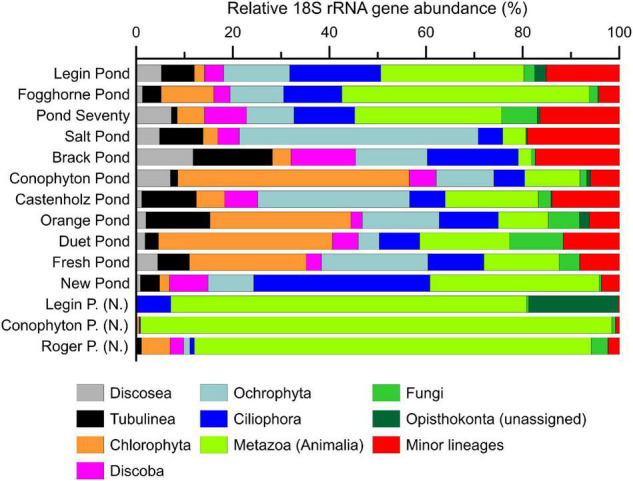
The relative abundance (%) of eukaryotic 18S rRNA genes in microbial mat communities. Shown are the major eukaryotic phyla. Detailed results from the 18S rRNA sequencing can be found in the supporting information. Samples with (N.) indicate ‘Nostoc spheres,’ which were collected in three different meltwater ponds.

The meltwater ponds contained several eukaryotic groups including, microalgae, amoebae, metazoa, and fungi (Figure 4 and Supplementary Tables 5–9). The ‘*Nostoc* sphere’ samples revealed a high relative abundance of rotifers compared to the other samples, suggesting that these microscopic animals are grazing on the cyanobacteria. In addition, these samples had a lower proportion of photosynthetic eukaryotes, which were primarily of the families Chlorophyceae and Bacillariophyceae. The other microbial mats from the meltwater ponds revealed a more heterogeneous distribution of eukaryotes, additionally featuring a higher proportion of protists such as the Amoebozoa (Discosea and Tubulinea) and ciliates. PCoA of the 18S rRNA gene sequences revealed no distinct eukaryal assemblages in the microbial mats from the meltwater ponds, consistent with previous observations that eukarya on the McMurdo Ice Shelf have a broad tolerance to the local environmental conditions (Jackson et al., 2021). The genetic diversity of phototrophic and heterotrophic eukaryotes in the microbial mats highlights that the meltwater ponds are a habitat for a diverse eukaryotic community. Protists, fungi, and microfauna vary in size and range from uni- to multicellular organisms. Therefore, it cannot be excluded that some methodological biases occur in the documented relative 18S rRNA gene abundance in the community composition.

### Source Assignments of Intact Polar Lipids in Meltwater Ponds

**FIGURE 5:**
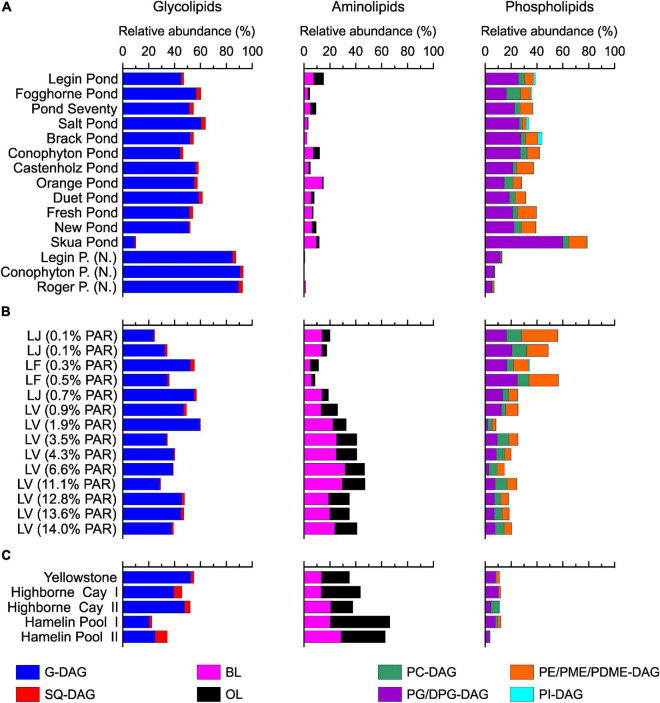
Intact polar lipids head group distribution in the meltwater ponds from the McMurdo Ice Shelf **(A)**. Samples with (N.) indicate ‘Nostoc spheres’, which were collected in three different meltwater ponds. Panel **(B)** shows the results from the three different Antarctic lakes: Lake Joyce (LJ), Lake Fryxell (LF), and Lake Vanda (LV). The brackets show the percentage of photosynthetically active radiation (PAR) that reached the microbial mats. The PAR was adopted from previous studies (Jungblut et al., 2016; Mackey et al., 2017b,^2018^; Matys et al., 2019). Panel **(C)** shows the relative abundance of IPLs in the microbial mats from the Yellowstone National Park (United States), Highborne Cay (Bahamas), and Hamelin Pool (Australia).

A variety of bacterial and eukaryal IPLs, comprising glycosidic, phosphatidic, and nitrogen-bearing head groups linked *via* ester bonds to the hydrophobic tail were detected in the meltwater ponds (Figure 5, for structures, see Supplementary Figure 4). In line with the molecular data (Supplementary Table 4), the results revealed a predominance of IPLs associated with cyanobacteria. Thus, glycolipids (G-DAGs), which are the major lipid class in cyanobacteria (Wada and Murata, 1998), showed the highest relative abundance (45 – 60%, Figure 5A). PG-DAG/DPG-DAG (di)-phosphoglycerol-DAG and SQ-DAG are also commonly detected in cyanobacteria (Wada and Murata, 1998) represented 14–25% and 1–3% of the total IPLs in the microbial mats from the McMurdo Ice Shelf (Figure 5A). Other detected IPLs, such as PC-DAG (2–11%), PE-DAG (phosphoethanolamine-DAG, 6–14%), and PI-DAG (∼1%), are rarely present in cyanobacteria (e.g., Wada and Murata, 1998), suggesting that these lipids are derived from other classes of bacteria or eukarya. This is also indicated by the 16S rRNA gene results, which showed that Proteobacteria and Bacteroidetes play an important role in the meltwater pond communities (Figure 2). PE-DAGs are ubiquitous in various bacterial groups (e.g., Goldfine and Hagen, 1968; Sturt et al., 2004), while PC-DAGs are commonly associated with eukarya (Raetz, 1986). Nevertheless, up to 15% of bacteria produce PC-DAGs (Geiger et al., 2013) – complicating the assignment of this lipid class to a specific biological source. Aminolipids were the least abundant lipids in the microbial mats from the meltwater ponds (2–15%, Figure 5A). Ornithine lipids, representing up to 8% of the total IPLs, are widespread in bacteria (Geiger et al., 2010), whereas betaine lipids (2–14%) are typically found in eukaryotic algae, fungi, and amoebae (Kato et al., 1996; Sohlenkamp et al., 2003), although they have been reported in cyanobacteria too (Rezanka et al., 2003). Archaeal IPLs were not detected in any mats from the meltwater ponds.

Principal coordinates analysis of the IPLs showed separate clusters of the ‘*Nostoc* spheres’ and the microbial mats (Figure 3). The IPL distribution in the ‘*Nostoc* spheres’ revealed a high abundance of G-DAGs (85 – 90%), while other IPLs were only present in trace amounts (Figure 5A). The IPL pattern agrees with previous results from *Nostoc* cultures (Bauersachs et al., 2009a; Schouten et al., 2013). Surprisingly, the *Nostoc commune* sheet in Fogghorne Pond did not cluster with the ‘*Nostoc* spheres’ as observed for the 16S rRNA gene analysis. Heterogenic distributions of the bacterial community in this mat may have resulted in variations between the DNA and IPL analysis because individual samples were collected for both types of analyses. An additional explanation may be that the different growth patterns, either as spheres (∼90% G-DAGs) or as sheets (∼55% G-DAGs), could affect the lipid composition in *Nostoc* communities. Future research is needed to investigate whether different growth patterns might affect the lipid composition in these microbes. Moreover, the PCoA showed no pronounced differences in the IPL distribution between fresh and brackish meltwater. This implies that the low to medium salt concentrations have little effect on the lipid composition of microbes in Antarctic meltwater ponds.

The microbial mat from the Skua Pond showed an IPL distribution distinct from the communities from the other meltwater ponds (Figure 5A). Here, phospholipids were predominant, in particular DPG-DAGs (47%) were abundant. The name ‘Skua’ arose from the presence of nesting skuas in the vicinity. Bird guano is possibly the source of elevated phosphorous in Skua Pond (Hawes et al., 2014). Alternatively, a distinct microbial community, which synthesizes high abundances of DPG-DAG seems also plausible given that this was the only sample that was collected as a broken and partially desiccated mat at the water line, while all other samples were subaqueous. Unfortunately, the Skua DNA samples were compromised during shipping, and thus we were unable to investigate 16S and 18S rRNA genes for this pond.

### Heterocyte Glycolipid Composition in McMurdo Ice Shelf Meltwater Ponds

**FIGURE 6:**
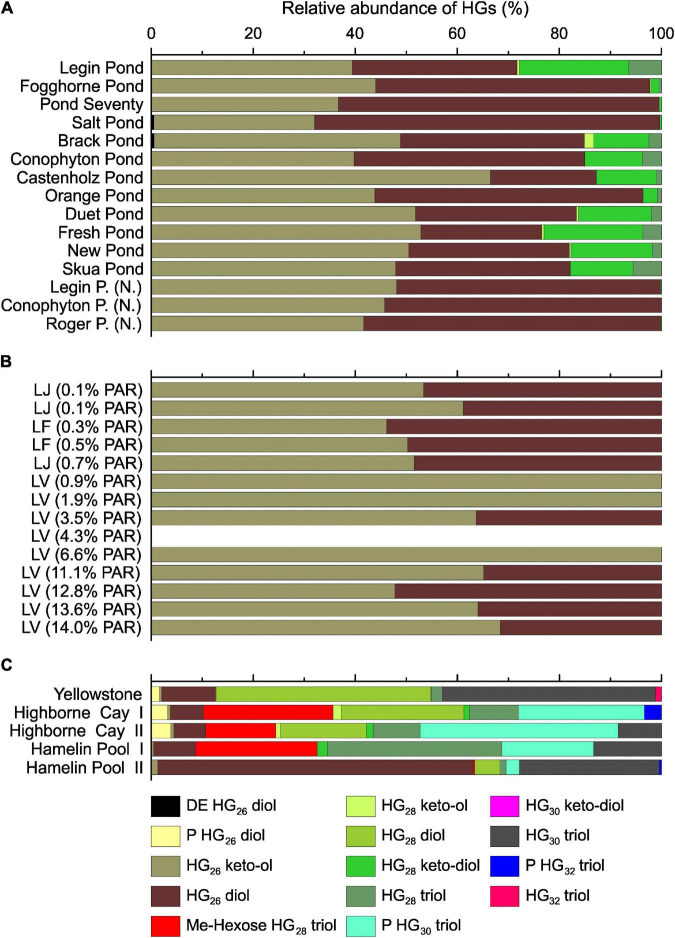
Heterocyte glycolipid distribution in the meltwater ponds from the McMurdo Ice Shelf **(A)**. Samples with (N.) indicate ‘Nostoc spheres,’ which were collected in three different meltwater ponds. Panel **(B)** shows the results from the three different Antarctic lakes: Lake Joyce (LJ), Lake Fryxell (LF) and Lake Vanda (LV). The brackets show the percentage of photosynthetically active radiation (PAR) that reached the microbial mats. The PAR was adopted from previous studies (Jungblut et al., 2016; Mackey et al., 2017b,^2018^; Matys et al., 2019). HGs in LV 4.3% PAR were below detection limit. Panel **(C)** shows the relative abundance of HGs in the microbial mats from the Yellowstone National Park (United States), Highborne Cay (Bahamas), and Hamelin Pool (Australia). DE, deoxyhexose; P, pentose; Me-hexose, methyl-hexose.

Seven different types of HGs were detected in the meltwater ponds (Figure 6, for structures, see Supplementary Figure 5). HG_26_ diols and keto-ols were most abundant and constituted between 70 and 100% of all HGs. Particularly, mats dominated by *Nostoc* spp. (‘*Nostoc* spheres’ and Fogghorne Pond) and *Nodularia* spp. (Salt Pond, Supplementary Table 1) showed a strong dominance of HG_26_ diols and keto-ols. This is in line with previous culture studies, in which both HGs were exclusively detected in these two species (e.g., Gambacorta et al., 1999; Bauersachs et al., 2009a). In addition, the predominance of HG_26_ diols and keto-ols in microbial mats from Pond Seventy suggests that the dominant but based on the 16S rRNA gene analysis enigmatic cyanobacterial species (Supplementary Table 1B), may be found with the Nostocaceae. The microbial mats from the other meltwater ponds, which showed a more heterogeneous distribution of cyanobacteria (Supplementary Table 1), contained 0 – 28% of HG_28_ diols, keto-diols, and triols. These HGs have been described as minor compounds in some Nostocaceae, such as *Anabaena* and *Aphanizomenon* spp. (Wörmer et al., 2012; Bauersachs et al., 2017), while they are predominant in representatives of Rivulariaceae, including *Calothrix* spp. (Bauersachs et al., 2009a).

### Bacteriohopanepolyol Composition in McMurdo Ice Shelf Meltwater Ponds

**FIGURE 7:**
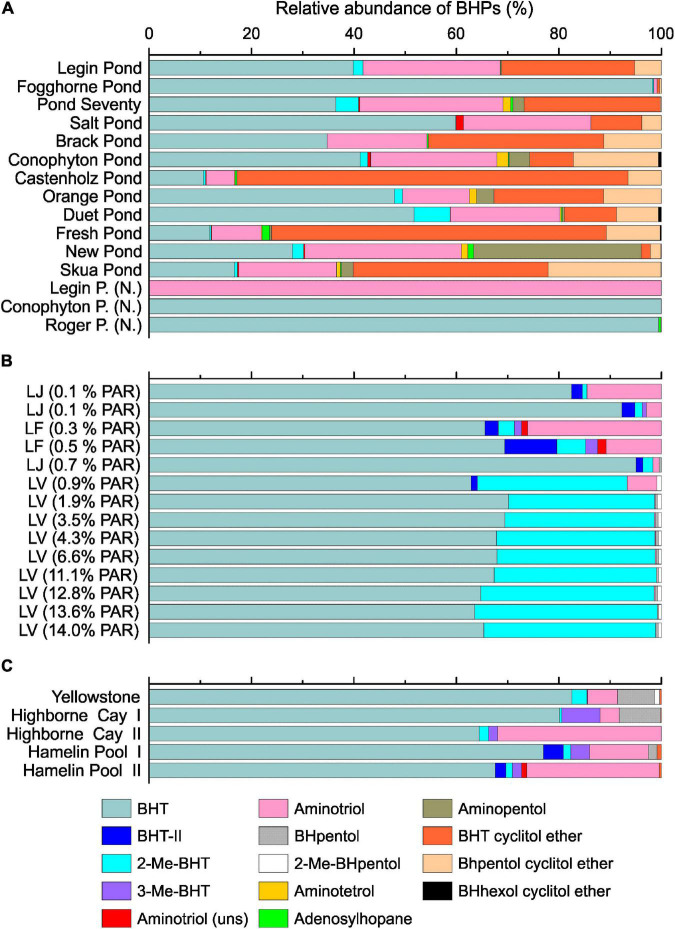
Bacteriohopanepolyols composition in the microbial mats from the meltwater ponds **(A)**. Samples with (N.) indicate ‘Nostoc spheres’ that were collected in three different meltwater ponds. Panel **(B)** shows the relative BHP distribution from the three different Antarctic lakes: Lake Joyce (LJ), Lake Fryxell (LF) and Lake Vanda (LV). The brackets show the percentage of photosynthetically active radiation (PAR) that reached the microbial mats. The PAR was adopted from previous studies (Jungblut et al., 2016; Mackey et al., 2017b,^2018^; Matys et al., 2019). Panel **(C)** shows the relative abundance of BHPs in the microbial mats from the Yellowstone National Park (United States), Highborne Cay (Bahamas), and Hamelin Pool (Australia).

The BHPs showed a heterogeneous distribution across the microbial mats and ‘*Nostoc* spheres’ (Figure 7, for structures, see Supplementary Figure 6). Accordingly, no significant connection between the microbial compositions and BHP distributions was visible. Bacteriohopanetetrol (BHT) was the predominant BHP in most samples, followed by aminotriol and BHT cyclitol ether. Microbial mats from Castenholz Pond, Fresh Pond, and Skua Pond, on the contrary, are dominated by BHT cyclitol ether over aminotriol, and BHT. All three components are detected in a wide range of bacteria, which precludes a clear biological assignment of these lipids (Talbot et al., 2008; Van Winden et al., 2012). Aminopentol, predominant in New Pond, has been suggested as a biomarker for methanotrophic bacteria type I (Van Winden et al., 2012), which is surprising given that these bacteria were not detected in this pond based on the 16S rRNA gene analysis. However, a recent study showed that nitrite-oxidizing bacteria are capable of synthesizing aminopentol (Elling et al., 2022), indicating that these compounds may be produced by a range of different bacteria and not exclusively by methanotrophs. Adenosylhopane, which has been reported from α- and β-Proteobacteria as well as cyanobacteria (e.g., Talbot et al., 2007; Van Winden et al., 2012), was a minor BHP in these mats (<2%).

2-Methylated BHPs (2-Me-BHPs) were only detected as minor compounds (<10%, Figure 7A) and, notably, were lacking in the three ‘*Nostoc* sphere’ samples. The low abundance of 2-Me-BHT is surprising given that these compounds were previously suggested to be broadly prevalent in cyanobacteria including some species of *Nostoc* (Summons et al., 1999; Doughty et al., 2009). It was subsequently proposed that expression of the *hpnP* protein responsible for the production of 2-Me-BHT in cyanobacteria is an environmental stress response (Kulkarni et al., 2013; Ricci et al., 2017). For instance, an increasing 2-Me-BHT content with decreasing light intensity was observed (Matys et al., 2019). The high light intensity during austral summer may thus be a factor in the observed low abundance of 2-Me-BHT in the meltwater ponds. In addition, since not all cyanobacteria produce methylated BHPs (Talbot et al., 2008), the taxonomic makeup of the cyanobacteria could lean toward those devoid of the *hpnP* gene. Moreover, the primacy of a predominantly cyanobacterial origin has diminished as other bacterial sources of 2-Me-BHP are discovered (Bisseret et al., 1985; Rashby et al., 2007; Ricci et al., 2015; Elling et al., 2020).

### Comparison of the Lipid Composition From Bacterial Mats Growing Under Different Environmental Conditions

#### Implications of Nutrient Supply on the Intact Polar Lipid Composition

**FIGURE 8:**
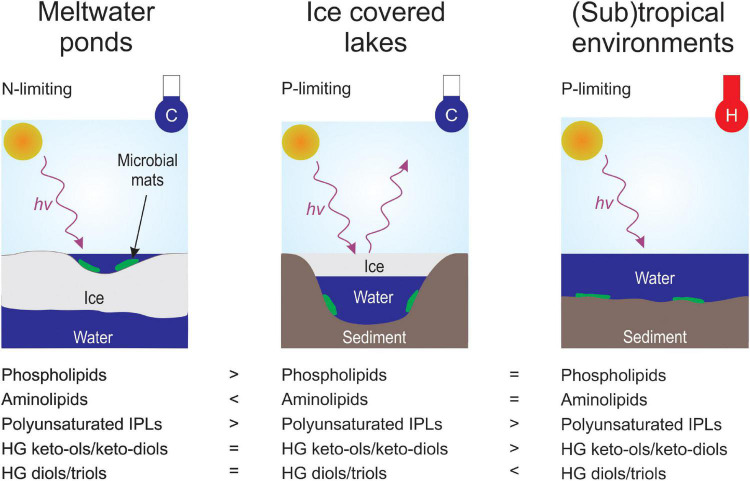
Conceptual sketch of the presenting controlling environmental parameters and the corresponding lipid membrane signatures. Environmental parameters include light regime (hv^+^), temperature in cold (C; blue thermometer) and warm (H; red thermometer), ice coverage and nutrient (N-limiting or P-limiting) availability.

The subtropical, hot spring, and Antarctic lake microbial mats showed a predominance of G-DAGs over other IPL-groups (20 – 60%), indicating that cyanobacteria likely play an important role in these settings. This agrees with previous DNA based studies showing that cyanobacteria are among the most abundant bacteria in Highborne Cay, Shark Bay, and the investigated Antarctic lakes (Dolhi et al., 2015; Mackey et al., 2015; Ruvindy et al., 2015; Matys et al., 2019). However, the microbial mats from (sub)tropical environments and Lake Vanda revealed a higher relative abundance of amino lipids (25–65%), such as BLs and OLs, than the mats from the meltwater ponds (5–15%, Figure 5). One explanation for the different abundance of amino and phospholipids between these sites is feasibly related to the availability of nitrogen and phosphorous. Low *N*/*P* ratios, commonly between 1 and 3, have been observed in the meltwater ponds (Howard-Williams et al., 1990; Sorrell et al., 2013), and Howard-Williams and Hawes (2007) argued that these ponds are primarily nitrogen limited. In Lake Vanda, Highborne Cay, and Hamelin Pool, typically, *N*/*P*-ratios above 20 have been reported (Atkinson, 1987; Priscu et al., 1989; Jabro, 2008). This suggests that P may be the limiting nutrient compared to the meltwater ponds, as suggested for Lake Vanda (Dillon et al., 2020). A replacement with N-bearing lipids in phospholipid-containing bacteria has been observed in various studies under P-limited conditions [see review by Schubotz (2019)]. This suggests that the low availability of phosphate in Lake Vanda and the subtropical mats leads to a substitution of phosphatidyl IPLs by aminolipids (Figure 8). Genes for the substitution of nitrogen for phosphorous in membrane lipids have been, for instance, recently detected in microbial mats from Lake Vanda (Dillon et al., 2020).

#### Implications of Temperature on Intact Polar Lipid Composition

Besides the IPL head group composition, the properties of the core fatty acids were investigated. This analysis revealed that the acyl chains in the (sub)tropical microbial mats were more saturated than in samples from Antarctica (Supplementary Figure 3 and Supplementary Table 10). These observations agree with previous culture-based experiments, where the abundance of unsaturated fatty acids increased with decreasing growth temperature (e.g., Marr and Ingraham, 1962; Suutari et al., 1990; Gombos et al., 1994). In addition, a correlation between bacterial membrane permeability and the degree of saturation of fatty acids has been shown in various earlier studies (e.g., Van de Vossenberg et al., 1995; Murata and Los, 1997). This implies that fatty acid desaturation is an adaptive microbial response to cold environments (e.g., Marr and Ingraham, 1962; Suutari et al., 1990; Gombos et al., 1994) and helps cyanobacteria and other microbes to thrive under the extreme conditions of the Antarctic meltwater ponds and lakes (Figure 8).

Interestingly, Antarctic lake samples contained a higher degree of saturation for most IPL types compared to the microbial mats from the meltwater ponds (Supplementary Figure 3 and Supplementary Table 10). Since samples from both environments were collected at comparable *in situ* temperatures (Tables 1, 2), this indicates that additional factors, such as ice coverage, could affect the degree of unsaturation in the IPL acyl chain. The meltwater ponds mats are only ice-free during the height of austral summer but frozen during winter (Hawes et al., 2011). In contrast, Lakes Vanda, Joyce, and Fryxell are perennially covered by a thick layer of ice but remain liquid all year. Previous experiments observed an improved resistance to freezing among different bacterial genera when their cell membrane contained high quantities of unsaturated fatty acids (Goldberg and Eschar, 1977; Beal et al., 2001). Accordingly, microbes in the meltwater ponds may increase the extent of unsaturation to survive prolonged periods of freezing during austral winter.

Another potential factor could be the different light regimes of the Antarctic lakes and meltwater ponds. The microbial mats from the Antarctic lakes are habituated to low light intensities (PAR ∼0.1–14% incident irradiance, see Section “Materials and Methods”), whereas a higher median PAR of ∼45% was determined for 39 different meltwater ponds on the McMurdo Ice Shelf during austral summer (Pridmore et al., 1995). Therefore, we hypothesize that the high light intensity during austral summer in the meltwater ponds results in the preferred synthesis of polyunsaturated membrane lipids. This is supported by previous experiments with *Synechococcus* sp. PCC7942 which have shown that an increase in unsaturation of fatty acids enhanced the tolerance to grow under strong light intensity (Gombos et al., 1997). Furthermore, a recent study investigating lipid distributions on a millimeter-scale in microbial mats from Octopus spring suggested that unsaturated IPLs form the first barrier against strong light exposure (Wörmer et al., 2020). However, additional research is needed to fully understand the correlation between light exposure and lipid membrane composition (Figure 8).

#### Temperature Controls on Heterocyte Glycolipid and Bacteriohopanepolyol Composition

As observed with the IPLs, the HGs revealed substantial differences between the microbial mats from the Antarctic environments (meltwater ponds and lakes) compared to the (sub)tropical and hot spring samples (Figure 6). In general, the latter had a higher diversity of HGs and extended carbon chains compared to the Antarctic settings. However, a unique feature of the microbial mats from Antarctica is the high abundance of HG keto-ols and keto-diols, whereas (sub)tropical and hot spring microbial mats almost exclusively contained HG diols and triols (Figure 6). Similar observations have been made in cultured cyanobacteria, in which the proportion of HG diols has been found to increase with growth temperature (Bauersachs et al., 2014). The HDI_26_, a HG based proxy to reconstruct surface water temperatures (SWT) in lacustrine environments (Bauersachs et al., 2015, 2021), revealed substantial variations between the Antarctic meltwater ponds (HDI_26_: 0.24 – 0.68, SWT: –6 – 11°C) and lakes (HDI_26_: 0.32 – 0.54, SWT: – 5 – 5°C) compared to the (sub)tropical mats (HDI_26_: 0.90 – 0.97, SWT: 21 – 23°C). HG distribution patterns and the HDI_26_ thus express a significant climate-driven component, which may compensate for varying rates of oxygen diffusion into the heterocyte with temperature variation and provide cyanobacteria with an anaerobic microenvironment to allow for biological N_2_ fixation (Bauersachs et al., 2009a).

The microbial mats from the McMurdo Ice Shelf showed substantial variations of the BHP distribution pattern in the different meltwater ponds, which complicated the comparison with samples from other environments (Figure 7). In general, the mats from the meltwater ponds revealed a high relative abundance of cyclitol ethers, while these compounds were below the detection limit in most of the samples from the Antarctic lakes and (sub)tropical environments. High quantities of cyclitol ethers have previously been detected in microbial mats collected from the Kongsfjord near Svalbard (Rethemeyer et al., 2010). It was suggested that these compounds are essential to protect bacteria to grow under microbial stress, such as a low pH or growth with antibiotics (Schmerk et al., 2015). However, cyclitol ethers are detected in a wide range of bacteria, which precludes a clear biological assignment of these lipids (Talbot et al., 2008; Van Winden et al., 2012; Schmerk et al., 2015).

Moreover, some noteworthy distribution patterns were observed in the microbial mats from Antarctic lakes and (sub)tropical environments. The microbial mats from Lake Vanda showed a high abundance of 2-Me-BHPs (30 – 35%). Such a high abundance of methylated BHPs was not detected in any other investigated environment. Based on genetic analysis of the radical *S*-adenosylmethionine protein that catalyzes the methylation of hopanoids at the C-position, a recent study suggested that 2-Me-BHPs in Lake Vanda are produced by cyanobacteria (Matys et al., 2019). Given that cyanobacteria dominated the microbial community in the McMurdo meltwater ponds and the low abundance of 2-Me-BHPs in these mats, this suggests that the synthesis of 2-Me-hopanoids is either dependent on the cyanobacterial taxon or particular environmental conditions. Moreover, we detected some BHPs in the Antarctic lakes and (sub)tropical environments that were absent from the meltwater ponds. BHpentol and 2-Me-BHpentol, which are considered biomarkers for cyanobacteria (Talbot et al., 2008), were detected in the mats from Lake Vanda, Yellowstone, and Highborne Cay, for instance. Moreover, 3-Me-BHT which has been suggested as a biomarker for methanotrophic bacteria (Zundel and Rohmer, 1985; Simonin et al., 1996) was detected in the mats from Lake Joyce, Lake Fryxell, Highborne Cay, and Hamelin Pool. This indicates that methanotrophic bacteria may be an important part of the microbial community in these samples.

Lastly, most of the samples investigated here came from shallow and highly productive environments which are expected to be fully saturated with O_2_. Samples from the Bratina meltwater ponds originated from waters that have previously been shown to be oversaturated (>625 μM) with respect to O_2_ (Wait et al., 2006). Moreover, Lake Vanda and Lake Joyce samples were collected in the O_2_ oversaturated upper part of the water column (Table 2). Thus, we did not observe trends in IPL composition that would have been influenced by O_2_ availability. On the other hand, mat samples from Lake Fryxell originated from an environment with a lower pO_2_ (Table 2), particularly the sample that was collected at 9.7 m depth (PAR 0.3%). This sample revealed a high relative abundance of BHT-II compared to the other investigated samples (Figure 7). BHT-II has been detected in marine anaerobic ammonium-oxidizing (anammox) bacteria (Rush et al., 2014), and freshwater purple non-sulfur bacteria (Neunlist et al., 1988) and it has been widely used to investigate oxygen gradients in the marine water column (e.g., Sáenz et al., 2011; Kharbush et al., 2013). Accordingly, the high abundance of BHT-II suggests the presence of an anaerobic bacterial community in the microbial mat from 9.7 m depth in Lake Vanda. This agrees with a recent study that showed a high abundance of genes indicative of anaerobic respiration capacity in microbial mats collected at a depth of 9.8 m in Lake Vanda (Dillon et al., 2020). However, we note that Rush et al. (2014) identified BHT-II only in marine and not freshwater anammox bacteria. Thus, salinity-stratified non-marine environments such as Lake Fryxell appear to also host bacterial sources of this unique BHP.

## Conclusion

The microbial mat communities in twelve meltwater ponds from the McMurdo Ice Shelf were investigated for their IPL, HG, and BHP contents and compared with 16S and 18S rRNA gene community compositions. IPL distribution patterns dominated by glycolipids are in accordance with the predominance of cyanobacteria in these environments. Our results revealed a high abundance of phospholipids in microbial mats from the meltwater ponds, whereas aminolipids substitute these in P-limited systems. Comparison with microbial mats in perennially ice-covered Antarctic lakes, (sub)tropical, and hot spring environments revealed that the microbes in the meltwater ponds produce highly unsaturated lipids likely to increase the fluidity of the lipid membrane in cold environments and protect the inner cell against high levels of solar irradiance during austral summer. Similarly, HGs produced by N_2_-fixing heterocytous cyanobacteria show unusually high relative proportions of HG keto-ols and keto-diols compared to cyanobacterial mats from (sub)tropical and hot spring environments; likely an adaption at low temperatures to maintain the integrity of the gas diffusion barrier and limit the inflow of O_2_ into the heterocyte.

## Data Availability Statement

The datasets presented in this study can be found in online repositories. The names of the repository/repositories and accession number(s) can be found in the article/Supplementary Material.

## Author Contributions

RES, IH, and TWE designed the research. TJM, IH, and RES collected samples. TWE, MJK, ADJ, TB, and TJM performed the research. TWE, ADJ, JLM, TB, and HG analyzed the data. TWE and RES wrote the manuscript with contributions from all co-authors. All authors contributed to the article and approved the submitted version.

## Conflict of Interest

The authors declare that the research was conducted in the absence of any commercial or financial relationships that could be construed as a potential conflict of interest.

## Publisher’s Note

All claims expressed in this article are solely those of the authors and do not necessarily represent those of their affiliated organizations, or those of the publisher, the editors and the reviewers. Any product that may be evaluated in this article, or claim that may be made by its manufacturer, is not guaranteed or endorsed by the publisher.
