# Supplementary material for: Lipid Biomarkers From Microbial Mats on the McMurdo Ice Shelf, Antarctica: Signatures for Life in the Cryosphere
Source: Front Microbiol. 2022 Jun 10;13:903621. doi: 10.3389/fmicb.2022.903621 (PMC9232131; doi:10.3389/fmicb.2022.903621)
Supplement: Supplementary file 1 [file Table_1.DOCX]

## Supporting information for

##

# **Lipid biomarkers from microbial mats on the McMurdo Ice Shelf, Antarctica: signatures for life in the cryosphere**

Content of this file

Figures S1 to S6

Tables S1 to S10

References


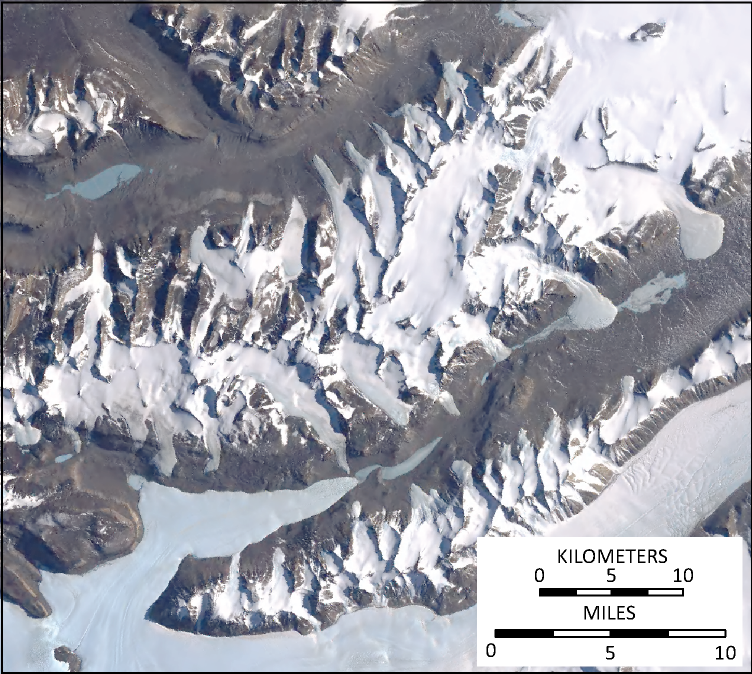


Lake Vanda

Lake Fryxell

Lake Joyce

*Fig. S1. The sampling location of in the three investigated perennially covered lakes in the McMurdo Dry Valleys (Lake Vanda, Lake Fryxell and Lake Joyce). Lakes are marked by red boxes. Samples were collected as described previously* (Hawes et al., 2011; Jungblut et al., 2016; Mackey et al., 2017)*.*


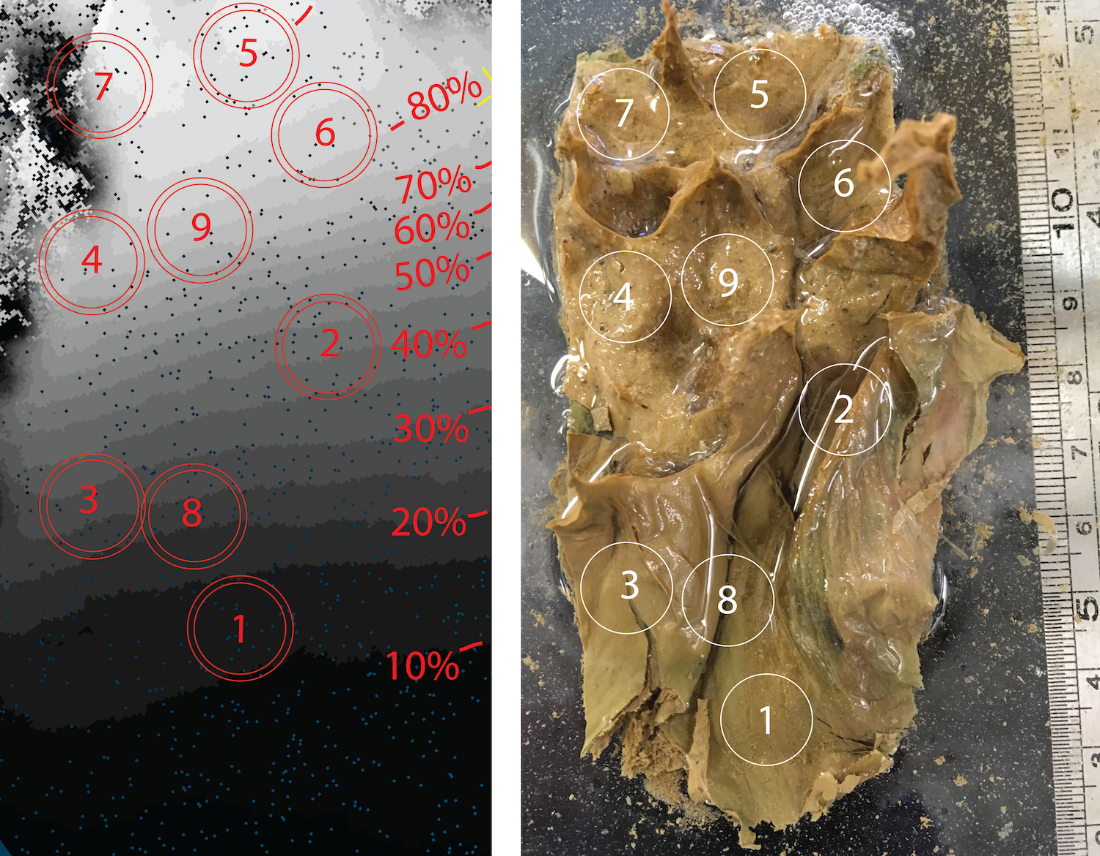


Fig. S2. Lake Vanda microbial mat subsampling sites for lipid biomarker analysis. Left) modeled PAR incident on microbial mat due to overhanging rocks on the lakebed. PAR is given as percent of ambient irradiance, modeled from 3D lake bed Structure from Motion reconstruction and measured angular light field (Mackey et al., 2017). The percent PAR listed in figures 4-6 is calculated from the percent ambient PAR at 9 m depth in Lake Vanda multiplied by the fraction of that irradiance received at each subsample site. Right) microbial mat showing the distribution of subsampling sites relative to microbial mat surface texture. The scale in both images is the same; slight differences in the orientation of subsample sites is due to deformation of the mat after thawing.

*Fig. S3.*

*
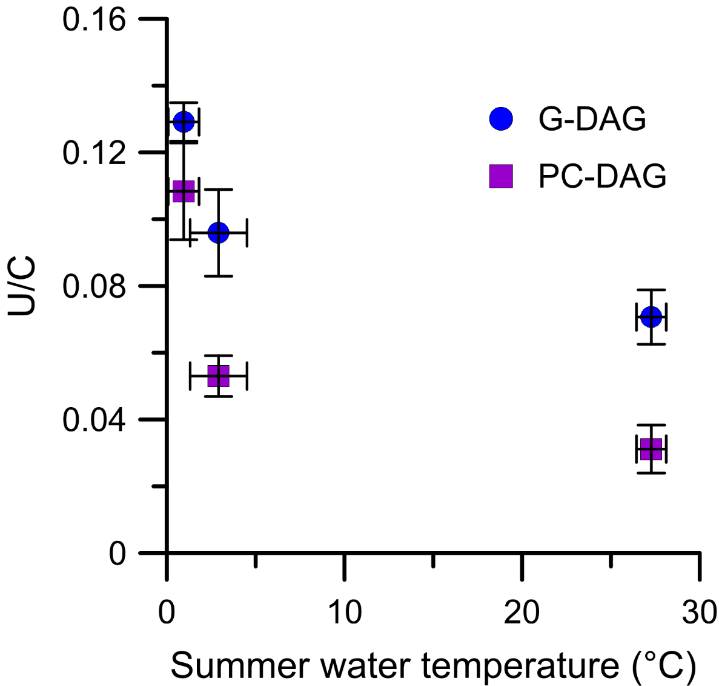
*

*Fig. S3. The average unsaturation per carbon atom (U/C) in G-DAG and PC-DAG in the microbial mats from McMurdo Ice Shelf meltwater ponds, Antarctic lake samples, and tropical environments. The vertical error bars represent the standard deviation for the unsaturations per C-atom for the individual data set, and the horizontal error bars represent the standard deviation of the mean in-situ/summer water temperature. For meltwater ponds and Antarctic lakes, temperatures are given in Tables 1 and 2. For Highborne Cay and Hamelin Pool, summer temperatures were compiled from Bowlin et al. (2012) and Suosaari et al. (2016). The sample from Yellowstone was not included since summer water temperature could not be estimated.*

*
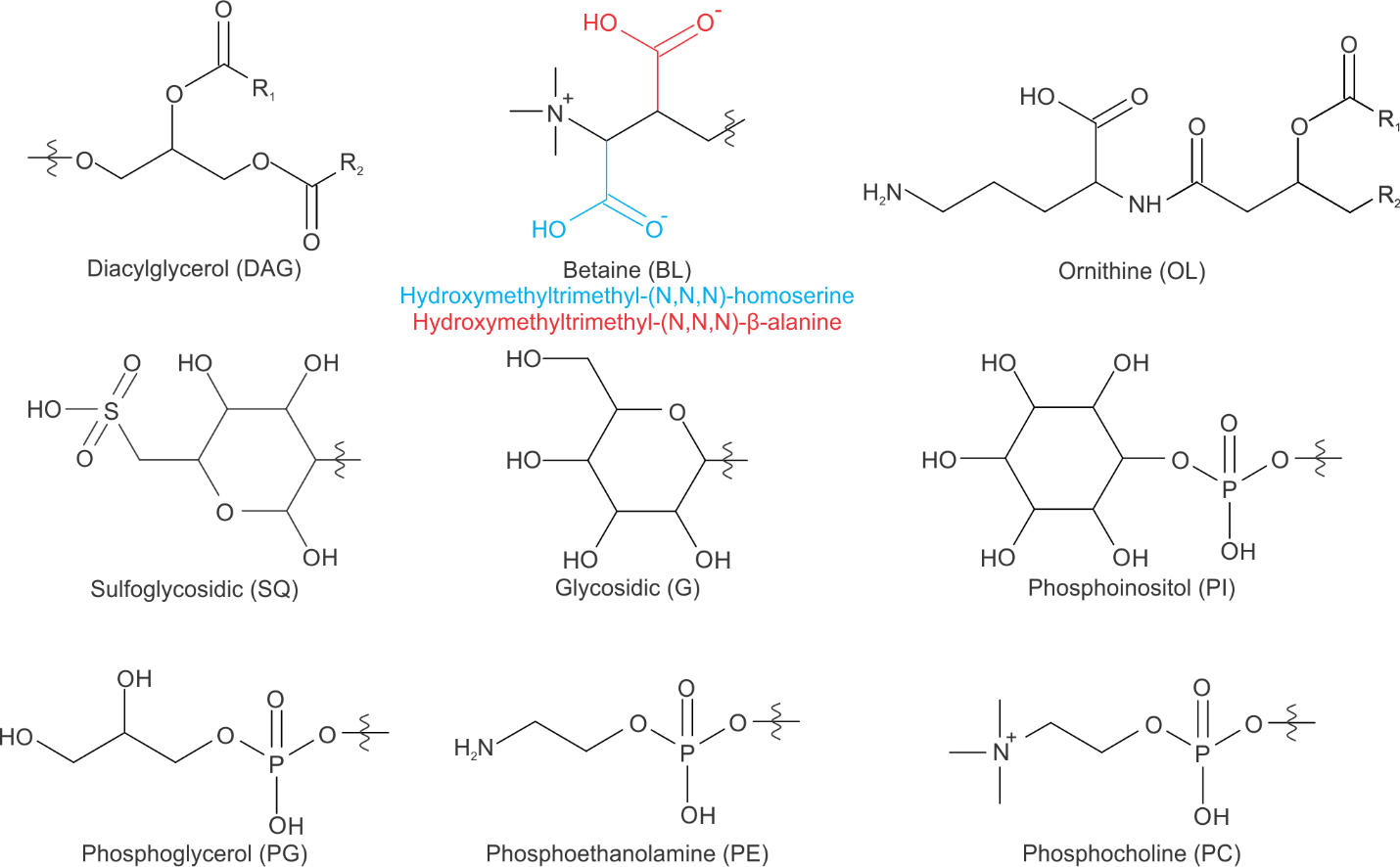
*

*Fig. S4. Intact polar lipid headgroups identified in this study*

*
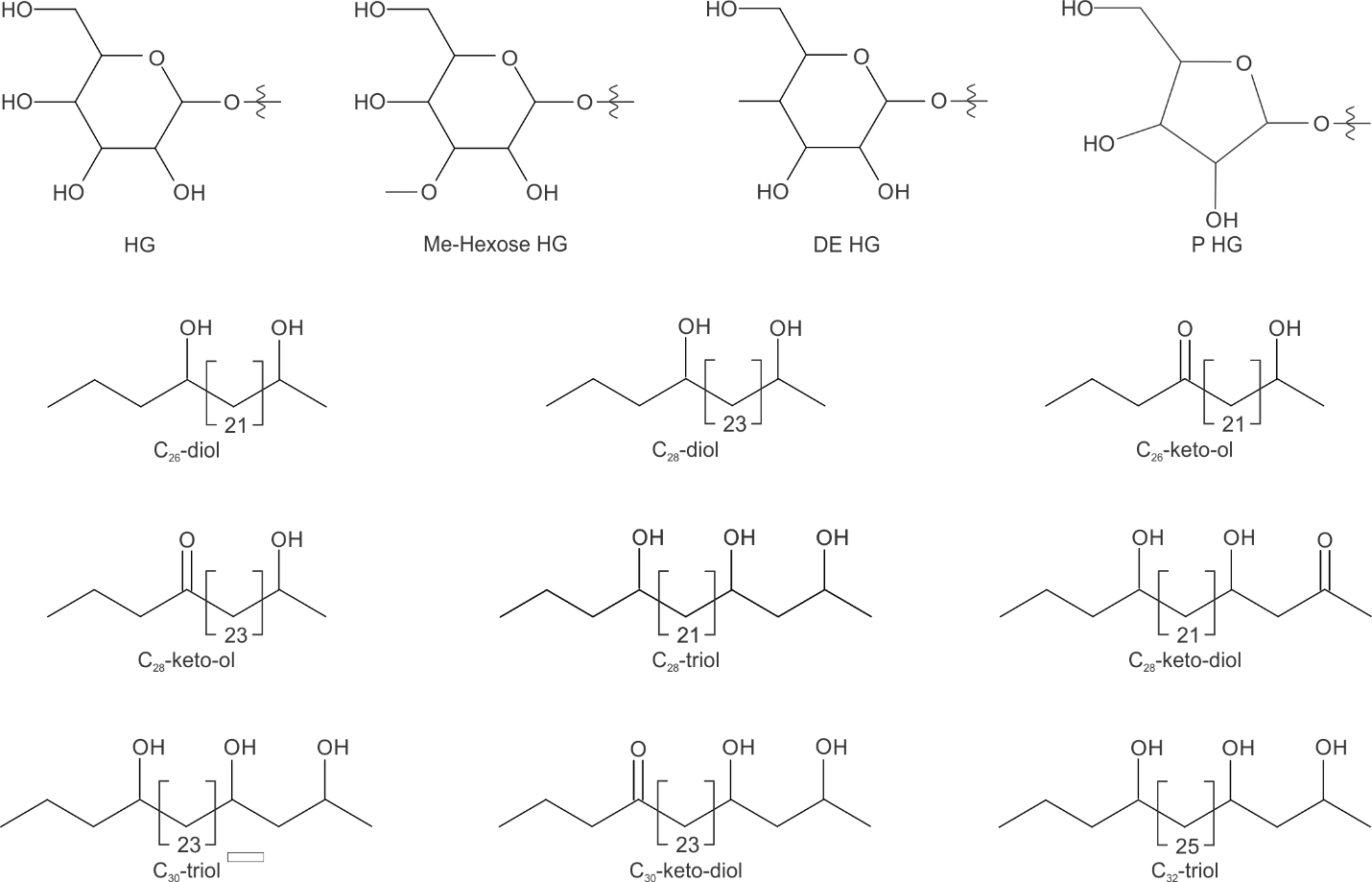
*

*Fig. S5. Heterocyte glycolipid structures identified in this study.*


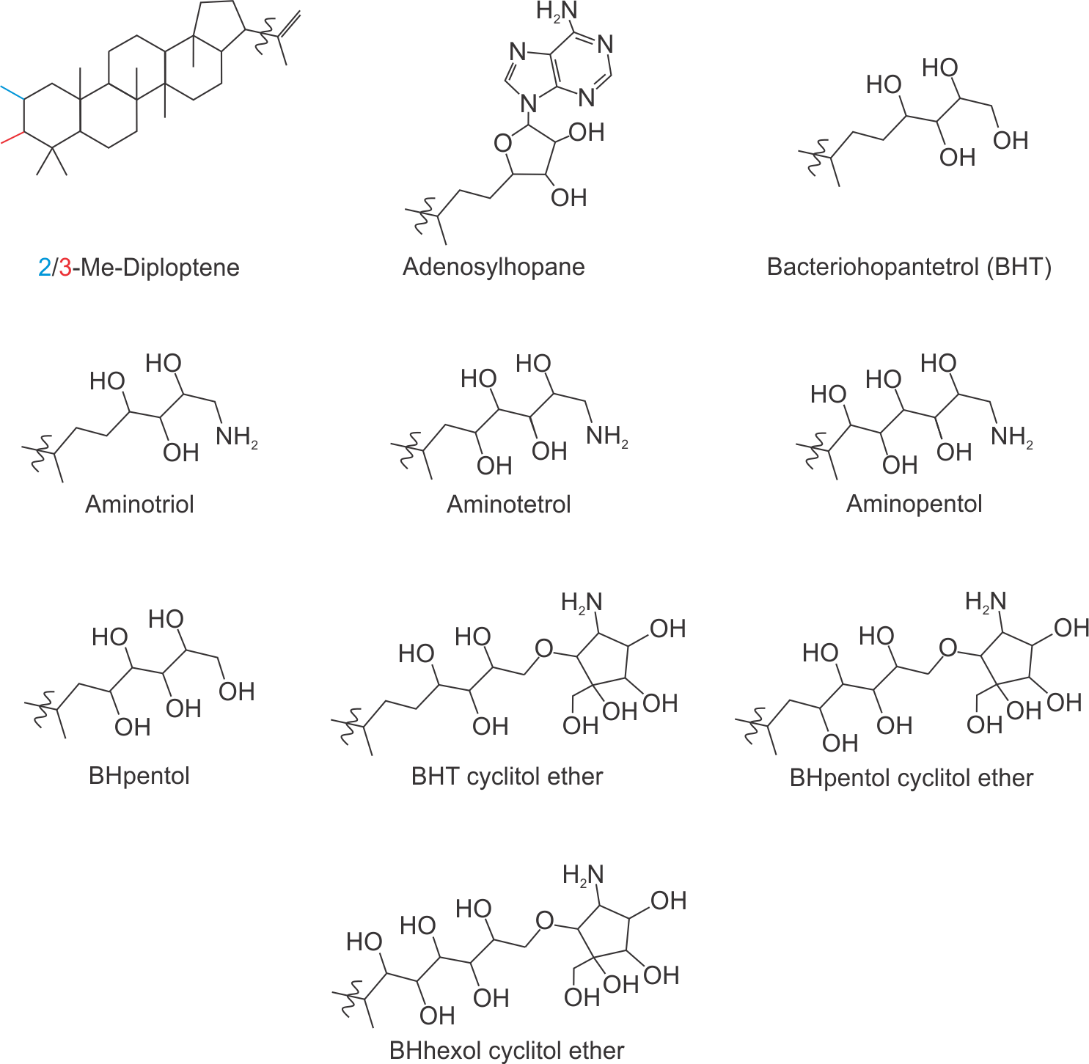


*Fig. S6. Bacteriohopanepolyol structures identified in this study.*

*Table S1a. Detected bacterial 16S rRNA sequences assigned with the phylum Cyanobacteria (Synechococcales and Oscillatoriales) with at least 1000 gene sequences in one sample. Shown are the average values of the replicates (n=3).*

| Class | Cyanobacteria | | | | | |
| --- | --- | --- | --- | --- | --- | --- |
| Order | Synechococcales | | Oscillatoriales | | | |
| Family | Leptolyngbyaceae | Pseudanabaenaceae | Coleofasciculaceae | | Oscillatoriaceaea | Phormidiaceae |
| Genus (strain) |  | *Pseudanabaena sp. (PCC-7429)* | *Wilmottia sp. (Ant-Ph58)* | *Geitlerinema sp.* | *Oscillatoria sp. (PCC-6304)* | *Tychonema sp. (CCAP 1459-11B)* |
| Sample | Average number of sequences | | | | | |
| Legin Pond | 28 | 674 | 500 | 1984 | 2 | 578 |
| Fogghorne Pond | 69 | 1204 | 3 | 29 | 0 | 296 |
| Pond Seventy | 12 | 1113 | 44 | 2051 | 285 | 759 |
| Salt Pond | 0 | 0 | 21 | 0 | 4388 | 0 |
| Brack Pond | 30 | 61 | 20 | 1207 | 7244 | 1095 |
| Conophyton Pond | 294 | 168 | 5196 | 1865 | 24 | 946 |
| Castenholz Pond | 52 | 1227 | 128 | 3984 | 19 | 1783 |
| Orange Pond | 818 | 6646 | 15 | 1595 | 0 | 1421 |
| Duet Pond | 327 | 2274 | 80 | 1491 | 8 | 1795 |
| Fresh Pond | 73 | 6563 | 0 | 3610 | 1 | 736 |
| New Pond | 2303 | 1488 | 0 | 1158 | 0 | 294 |
| Legin P. (N.) | 20 | 22 | 0 | 216 | 0 | 24 |
| Conophyton P. (N.) | 3 | 0 | 33 | 22 | 1742 | 68 |
| Roger P. (N.) | 807 | 177 | 11 | 631 | 3 | 9 |

Table S1b. Detected bacterial 16S rRNA sequences assigned with the phylum Cyanobacteria (Nostococales and Phormidesmiales with at least 1000 gene sequences in one sample. Shown are the average values of the replicates (n=3).

| Class | Cyanobacteria | | | | |
| --- | --- | --- | --- | --- | --- |
| Order | Nostococales | | | unknown | Phormidesmiales |
| Family | Aphanizomenonaceae | | Nostocaceae | unknown | Nodosilineaceae |
| Genus (strain) | *Aphanizomenon sp. (NIES81)* | *Nodularia sp. (PCC-9350)* | *Nostoc sp. (PCC-73102)* | unknown | *Nodosilinea sp. (PCC-7104)* |
| Sample | Average number of sequences | | | | |
| Legin Pond | 302 | 355 | 39 | 3687 | 526 |
| Fogghorne Pond | 509 | 225 | 9696 | 7 | 22 |
| Pond Seventy | 14 | 1215 | 28 | 4961 | 1188 |
| Salt Pond | 0 | 11936 | 23 | 0 | 750 |
| Brack Pond | 104 | 372 | 2 | 222 | 828 |
| Conophyton Pond | 2 | 233 | 194 | 2399 | 78 |
| Castenholz Pond | 391 | 1694 | 102 | 1400 | 210 |
| Orange Pond | 252 | 278 | 157 | 546 | 475 |
| Duet Pond | 218 | 875 | 140 | 2298 | 64 |
| Fresh Pond | 3109 | 1094 | 2 | 592 | 15 |
| New Pond | 92 | 253 | 32 | 824 | 120 |
| Legin P. (N.) | 1 | 7 | 15649 | 54 | 0 |
| Conophyton P. (N.) | 0 | 62 | 11688 | 12 | 0 |
| Roger P. (N.) | 1 | 7 | 14394 | 163 | 25 |

Table S2. Detected bacterial 16S rRNA sequences assigned with the phylum Proteobacteria with at least 500 gene sequences in one sample. Shown are the average values of the replicates (n=3).

| Class | Alphaproteobacteria | | Betaproteobacteria |
| --- | --- | --- | --- |
| Order | Rhodobacterales | Sphingomonadales | Burkholderiales |
| Familiy | Rhodobacteraceae | Sphingomonadaceae | Comamonadaceae |
| Genus (strain) | unknown | *Blastomonas* | *Hydrogenophaga* |
| Sample | Average number of sequences | | |
| Legin Pond | 427 | 13 | 815 |
| Fogghorne Pond | 1810 | 109 | 1681 |
| Pond Seventy | 1309 | 1 | 560 |
| Salt Pond | 754 | 0 | 51 |
| Brack Pond | 1529 | 1 | 668 |
| Conophyton Pond | 608 | 6 | 595 |
| Castenholz Pond | 380 | 11 | 1340 |
| Orange Pond | 847 | 12 | 1093 |
| Duet Pond | 243 | 0 | 979 |
| Fresh Pond | 186 | 0 | 418 |
| New Pond | 365 | 5 | 752 |
| Legin P. (N.) | 3510 | 577 | 3465 |
| Conophyton P. (N.) | 5899 | 11 | 5041 |
| Roger P. (N.) | 1569 | 89 | 3516 |

Table S3. Detected bacterial 16S rRNA sequences assigned with the phylum Bacteroidetes with at least 500 gene sequences in one sample. Shown are the average values of the replicates (n=3).

| Class | Sphingobacteriia | | | | | Bacteroidia | |
| --- | --- | --- | --- | --- | --- | --- | --- |
| Order | Sphingobacteriales | | | | | Cytophagales | |
| Family | Chitinophagaceae | | Saprospiraceae | Cyclobacteriaceaea | | Microscillaceae | Microscillaceae |
| Genus (strain) | *Ferruginibacter* | unknown | unknown | *Algoriphagus* | unknown | unknown | *Flavobacterium* |
| Sample | Average number of sequences | | | | | | |
| Legin Pond | 648 | 310 | 84 | 341 | 1336 | 420 | 440 |
| Fogghorne Pond | 32 | 7 | 7 | 1012 | 47 | 0 | 2268 |
| Pond Seventy | 19 | 666 | 78 | 379 | 1413 | 2 | 859 |
| Salt Pond | 0 | 0 | 692 | 167 | 0 | 0 | 2 |
| Brack Pond | 0 | 174 | 186 | 386 | 91 | 0 | 321 |
| Conophyton Pond | 377 | 255 | 44 | 459 | 374 | 5 | 732 |
| Castenholz Pond | 204 | 920 | 22 | 328 | 775 | 709 | 499 |
| Orange Pond | 7 | 138 | 54 | 1152 | 475 | 0 | 749 |
| Duet Pond | 342 | 87 | 33 | 91 | 217 | 1615 | 271 |
| Fresh Pond | 268 | 52 | 21 | 244 | 291 | 533 | 577 |
| New Pond | 837 | 53 | 115 | 391 | 419 | 594 | 1199 |
| Legin P. (N.) | 2 | 2 | 1 | 8 | 21 | 0 | 159 |
| Conophyton P. (N.) | 0 | 4 | 0 | 0 | 0 | 0 | 122 |
| Roger P. (N.) | 4 | 41 | 2 | 421 | 74 | 0 | 941 |

Table S4. Detected archaeal and bacterial 16S rRNA gene sequences. Shown are the average values of the replicates (n=3).

|  | Archaea | Bacteria |
| --- | --- | --- |
| Sample | Average number of sequences | |
| Legin Pond | 0 | 25000 |
| Fogghorne Pond | 0 | 25000 |
| Pond Seventy | 2.3 | 24997.7 |
| Salt Pond | 0.5 | 24999.5 |
| Brack Pond | 0 | 25000 |
| Conophyton Pond | 0 | 25000 |
| Castenholz Pond | 0 | 25000 |
| Orange Pond | 0 | 25000 |
| Duet Pond | 0 | 25000 |
| Fresh Pond | 0 | 25000 |
| New Pond | 0 | 25000 |
| Legin P. (N.) | 0 | 25000 |
| Conophyton P. (N.) | 0 | 25000 |
| Roger P. (N.) | 0 | 25000 |

Table S5a. Detected eukaryal 18S rRNA sequences assigned with the SAR supergroup with at least 500 gene sequences in one sample. Shown are the average values of the replicates (n=3). Details represent the highest cultured match.

| Major Rank | SAR | | | | | | | |
| --- | --- | --- | --- | --- | --- | --- | --- | --- |
| Sub rank | Ochrophyta | | | | | | | unknown |
| Details | *Chrysophyceae* | *Chlamydomyxa* | *Poteriospumella* | *Sellaphora* | Bacillariophyceae | unknown | unknown | |
| Name | Average number of sequences | | | | | | | |
| Legin Pond | 114 | 0 | 69 | 41 | 1081 | 1444 | 446 | |
| Fogghorne Pond | 195 | 0 | 369 | 0 | 623 | 1382 | 3 | |
| Pond Seventy | 525 | 0 | 9 | 9 | 9 | 1734 | 1064 | |
| Salt Pond | 159 | 1302 | 0 | 5822 | 0 | 4694 | 546 | |
| Brack Pond | 563 | 0 | 24 | 390 | 712 | 1020 | 409 | |
| Conophyton Pond | 1279 | 0 | 7 | 38 | 71 | 1153 | 72 | |
| Castenholz Pond | 118 | 0 | 4863 | 120 | 566 | 1895 | 56 | |
| Orange Pond | 78 | 0 | 264 | 3 | 539 | 2296 | 9 | |
| Duet Pond | 205 | 0 | 147 | 0 | 119 | 479 | 129 | |
| Fresh Pond | 27 | 0 | 56 | 243 | 3442 | 1596 | 100 | |
| New Pond | 57 | 0 | 138 | 6 | 419 | 1286 | 2 | |
| Legin P. (N.) | 0 | 0 | 0 | 0 | 4 | 6 | 0 | |
| Conophyton P. (N.) | 0 | 0 | 0 | 0 | 0 | 24 | 0 | |
| Roger P. (N.) | 1 | 0 | 87 | 4 | 26 | 159 | 8 | |

Table S5b. Detected eukaryal 18S rRNA sequences assigned with the SAR supergroup with at least 500 gene sequences in one sample. Shown are the average values of the replicates (n=3). Details represent the highest cultured match.

| Major Rank | SAR | | | | | | |  |
| --- | --- | --- | --- | --- | --- | --- | --- | --- |
| Sub rank | Ciliophora | | | | | | Bicosoecida | Stramenopiles |
| Details | *Euplotes* | *Hemiurosomoida* | *Cyrtophoria* | *Hypotrichia* | *Peniculia* | unknown | Cafeteria | unknown |
| Name | Average number of sequences | | | | | | | |
| Legin Pond | 435 | 422 | 228 | 844 | 564 | 1277 | 0 | 543 |
| Fogghorne Pond | 0 | 157 | 186 | 508 | 1414 | 181 | 0 | 384 |
| Pond Seventy | 295 | 907 | 35 | 637 | 60 | 520 | 0 | 633 |
| Salt Pond | 0 | 0 | 0 | 459 | 0 | 41 | 696 | 1616 |
| Brack Pond | 0 | 2186 | 0 | 993 | 25 | 447 | 0 | 1525 |
| Conophyton Pond | 62 | 269 | 24 | 405 | 353 | 237 | 21 | 577 |
| Castenholz Pond | 316 | 205 | 901 | 137 | 118 | 169 | 0 | 951 |
| Orange Pond | 125 | 358 | 248 | 297 | 1395 | 150 | 0 | 739 |
| Duet Pond | 120 | 178 | 466 | 740 | 146 | 271 | 0 | 162 |
| Fresh Pond | 65 | 146 | 1317 | 932 | 107 | 257 | 0 | 253 |
| New Pond | 1836 | 831 | 287 | 4637 | 675 | 729 | 0 | 148 |
| Legin P. (N.) | 0 | 0 | 252 | 0 | 0 | 547 | 0 | 5 |
| Conophyton P. (N.) | 0 | 34 | 0 | 0 | 0 | 0 | 0 | 14 |
| Roger P. (N.) | 59 | 20 | 104 | 12 | 0 | 0 | 0 | 224 |

Table S6a. Detected eukaryal 18S rRNA sequences assigned with the Opisthokonta supergroup with at least 500 gene sequences in one sample. Shown are the average values of the replicates (n=3). Details represent the highest cultured match.

| Major Rank | Opisthokonta | | | |
| --- | --- | --- | --- | --- |
| Sub rank | Bdelloidea | Chromadorea | Eutardigrada | Rhabdocoela |
| Details | *Adinetida* | *Araeolaimida* | *Parachela* | *Neodalyellida* |
| Name | Average number of sequences | | | |
| Legin Pond | 1607 | 1828 | 3324 | 487 |
| Fogghorne Pond | 12479 | 2 | 274 | 0 |
| Pond Seventy | 6973 | 0 | 312 | 0 |
| Salt Pond | 8 | 0 | 0 | 0 |
| Brack Pond | 39 | 0 | 0 | 0 |
| Conophyton Pond | 1737 | 142 | 719 | 0 |
| Castenholz Pond | 279 | 4342 | 65 | 0 |
| Orange Pond | 1866 | 13 | 357 | 159 |
| Duet Pond | 2501 | 1283 | 857 | 0 |
| Fresh Pond | 1734 | 424 | 540 | 1088 |
| New Pond | 2637 | 1158 | 4957 | 0 |
| Legin P. (N.) | 18371 | 0 | 0 | 0 |
| Conophyton P. (N.) | 24341 | 0 | 0 | 0 |
| Roger P. (N.) | 20477 | 0 | 48 | 0 |

Table S6b. Detected eukaryal 18S rRNA sequences assigned with the Opisthokonta supergroup with at least 500 gene sequences in one sample. Shown are the average values of the replicates (n=3). Details represent the highest cultured match.

| Major Rank | Opisthokonta | | | |
| --- | --- | --- | --- | --- |
| Sub rank | Metazoa (Animalia) | Fungi | | unknown |
| Details | unknown | *Cryptomycota* | unknown | unknown |
| Name | Average number of sequences | | | |
| Legin Pond | 143 | 42 | 453 | 603 |
| Fogghorne Pond | 23 | 118 | 321 | 60 |
| Pond Seventy | 79 | 585 | 1266 | 143 |
| Salt Pond | 1202 | 0 | 34 | 40 |
| Brack Pond | 509 | 80 | 69 | 12 |
| Conophyton Pond | 43 | 84 | 265 | 198 |
| Castenholz Pond | 30 | 67 | 337 | 49 |
| Orange Pond | 14 | 243 | 1370 | 496 |
| Duet Pond | 0 | 0 | 2678 | 0 |
| Fresh Pond | 75 | 2 | 987 | 30 |
| New Pond | 2 | 11 | 83 | 9 |
| Legin P. (N.) | 3 | 14 | 60 | 4655 |
| Conophyton P. (N.) | 11 | 0 | 131 | 6 |
| Roger P. (N.) | 0 | 0 | 606 | 3 |

Table S7. Detected eukaryal 18S rRNA sequences assigned with the Archaeplastida supergroup with at least 500 gene sequences in one sample. Shown are the average values of the replicates (n=3). Details represent the highest cultured match.

| Major Rank | Archaeplastida | | | |
| --- | --- | --- | --- | --- |
| Sub rank | Chlorophyceae | | | |
| Details | *Chlamydomonas sp. (ICE-W)* | *Chlamydomonas* | *Chlorococcales sp. (VII3)* | unassigned |
| Name | Average number of sequences | | | |
| Legin Pond | 55 | 27 | 158 | 260 |
| Fogghorne Pond | 151 | 62 | 58 | 2232 |
| Pond Seventy | 643 | 32 | 64 | 245 |
| Salt Pond | 734 | 0 | 0 | 15 |
| Brack Pond | 186 | 7 | 4 | 667 |
| Conophyton Pond | 5422 | 851 | 3853 | 1504 |
| Castenholz Pond | 490 | 30 | 182 | 305 |
| Orange Pond | 913 | 429 | 2890 | 2768 |
| Duet Pond | 289 | 34 | 787 | 7866 |
| Fresh Pond | 445 | 41 | 413 | 4237 |
| New Pond | 69 | 0 | 42 | 357 |
| Legin P. (N.) | 0 | 0 | 0 | 14 |
| Conophyton P. (N.) | 0 | 18 | 28 | 104 |
| Roger P. (N.) | 18 | 474 | 633 | 225 |

Table S8. Detected eukaryal 18S rRNA sequences assigned with the Amoebozoa supergroup with at least 500 gene sequences in one sample. Shown are the average values of the replicates (n=3). Details represent the highest cultured match.

| Major Rank | Amoebozoa | | | |
| --- | --- | --- | --- | --- |
| Sub rank | Discosea | | Tubulinea | |
| Details | *Platyamoeba* | *Vannella* | BOLA868 | Leptomyxida |
| Name | Average number of sequences | | | |
| Legin Pond | 641 | 379 | 1261 | 0 |
| Fogghorne Pond | 35 | 282 | 940 | 0 |
| Pond Seventy | 574 | 843 | 301 | 0 |
| Salt Pond | 858 | 0 | 0 | 2254 |
| Brack Pond | 1572 | 1373 | 3639 | 12 |
| Conophyton Pond | 797 | 948 | 165 | 0 |
| Castenholz Pond | 25 | 246 | 2796 | 0 |
| Orange Pond | 4 | 440 | 3206 | 0 |
| Duet Pond | 84 | 183 | 536 | 0 |
| Fresh Pond | 51 | 1034 | 1287 | 0 |
| New Pond | 90 | 119 | 994 | 0 |
| Legin P. (N.) | 2 | 0 | 4 | 0 |
| Conophyton P. (N.) | 0 | 0 | 0 | 0 |
| Roger P. (N.) | 6 | 7 | 232 | 0 |

Table S9. Detected eukaryal 18S rRNA sequences assigned with the Excavata supergroup with at least 500 gene sequences in one sample. Shown are the average values of the replicates (n=3). Details represent the highest cultured match.

| Major Rank | Excavata | | | |
| --- | --- | --- | --- | --- |
| Sub rank | Discoba | | | |
| Details | *Tetramitia* | *Neobodo* | *Rhynchomonas* | *Discicristata* |
| Name | Average number of sequences | | | |
| Legin Pond | 34 | 710 | 270 | 275 |
| Fogghorne Pond | 54 | 537 | 388 | 149 |
| Pond Seventy | 603 | 610 | 158 | 408 |
| Salt Pond | 9 | 1119 | 316 | 61 |
| Brack Pond | 1052 | 1655 | 745 | 608 |
| Conophyton Pond | 666 | 485 | 137 | 86 |
| Castenholz Pond | 133 | 1542 | 416 | 797 |
| Orange Pond | 320 | 141 | 0 | 141 |
| Duet Pond | 59 | 1140 | 588 | 552 |
| Fresh Pond | 10 | 781 | 427 | 354 |
| New Pond | 68 | 1923 | 165 | 914 |
| Legin P. (N.) | 0 | 0 | 0 | 0 |
| Conophyton P. (N.) | 0 | 6 | 6 | 0 |
| Roger P. (N.) | 661 | 44 | 0 | 9 |

Table S10. Calculated unsaturations per carbon atom for the different IPL head groups from the microbial mats from the Meltwater ponds, Antarctic lakes and (Sub)tropical environments. Values represent the average and standard deviation (in brackets) for the different environments. Note: due to unclear temperature conditions in the mat from Yellowstone, this sample was excluded from this table.

| IPL headgroup | Meltwater ponds | Antarctic lakes | (Sub)tropical environments |
| --- | --- | --- | --- |
| G-DAG | 0.129 (±0.006) | 0.096 (±0.013) | 0.071 (±0.08) |
| SQ-DAG | 0.067 (±0.005) | 0.049 (±0.012) | 0.033 (±0.007) |
| BL | 0.076 (±0.014) | 0.049 (±0.009) | 0.037 (±0.021) |
| OL | 0.057 (±0.018) | 0.041 (±0.006) | 0.011 (±0.008) |
| PC-DAG | 0.108 (±0.014) | 0.053 (±0.006) | 0.031 (±0.007) |
| PG/DPG-DAG | 0.062 (±0.009) | 0.038 (±0.006) | 0.0034 (±0.01) |
| PE/PME/PDME-DAG | 0.058 (±0.01) | 0.043 (±0.007) | 0.022 (±0.014) |
| PI-DAG | 0.062 (±0.024) | 0.029 (±0.008) | 0.063 (±0.053) |

## References

Dixon, P. (2003). VEGAN, a package of R functions for community ecology. *J. Veg. Sci.* 14, 927–930.

Hawes, I., Sumner, D. Y., Andersen, D. T., and Mackey, T. J. (2011). Legacies of recent environmental change in the benthic communities of Lake Joyce, a perennially ice-covered Antarctic lake. *Geobiology* 9, 394–410.

Jungblut, A. D., Hawes, I., Mackey, T. J., Krusor, M., Doran, P. T., Sumner, D. Y., et al. (2016). Microbial mat communities along an oxygen gradient in a perennially ice-covered Antarctic lake. *Appl. Environ. Microbiol.* 82, 620–630.

Mackey, T. J., Sumner, D. Y., Hawes, I., and Jungblut, A. D. (2017). Morphological signatures of microbial activity across sediment and light microenvironments of Lake Vanda, Antarctica. *Sediment. Geol.* 361, 82–92.

R Core Team (2021). A language and environment for statistical computing.
